# Next-generation sequencing identifies contribution of both class I and II HLA genes on susceptibility of multiple sclerosis in Japanese

**DOI:** 10.1186/s12974-019-1551-z

**Published:** 2019-08-05

**Authors:** Kotaro Ogawa, Tatsusada Okuno, Kazuyoshi Hosomichi, Akiko Hosokawa, Jun Hirata, Ken Suzuki, Saori Sakaue, Makoto Kinoshita, Yoshihiro Asano, Katsuichi Miyamoto, Ituro Inoue, Susumu Kusunoki, Yukinori Okada, Hideki Mochizuki

**Affiliations:** 1 Department of Statistical Genetics, Osaka University Graduate School of Medicine, 2-2 Yamadaoka, Suita, 565-0871 Japan; 2 Department of Neurology, Osaka University Graduate School of Medicine, Suita, 565-0871 Japan; 3 Department of Bioinformatics and Genomics Graduate School of Advanced Preventive Medical Sciences, Kanazawa University, Kanazawa, 920-8640 Japan; 4 Department of Neurology, Suita Municipal Hospital, Suita, 564-8567 Japan; 5 Pharmaceutical Discovery Research Laboratories, Teijin Pharma Limited, Hino, 191-8512 Japan; 6 Department of Cardiovascular Medicine, Osaka University Graduate School of Medicine, Suita, 565-0871 Japan; 7 Department of Neurology, Kindai University Faculty of Medicine, Osaka-Sayama, 589-8511 Japan; 8 Division of Human Genetics, National Institute of Genetics, Shizuoka, 411-8540 Japan; 9 Laboratory of Statistical Immunology, Immunology Frontier Research Center (WPI-IFReC), Osaka University, Suita, 565-0871 Japan

**Keywords:** Multiple sclerosis, Neuromyelitis optica spectrum disorder, HLA, Next-generation sequencing

## Abstract

**Background:**

The spectrum of classical and non-classical HLA genes related to the risk of multiple sclerosis (MS) and neuromyelitis optica spectrum disorder (NMOSD) in the Japanese population has not been studied in detail. We conducted a case-control analysis of classical and non-classical HLA genes.

**Methods:**

We used next-generation sequencing (NGS)-based HLA genotyping methods for mapping risk for 45 MS patients, 31 NMOSD patients, and 429 healthy controls. We evaluated the association of the HLA variants with the risk of MS and NMOSD using logistic regression analysis and Fisher’s exact test.

**Results:**

We confirmed that HLA-DRB1*15:01 showed the strongest association with MS (*P* = 2.1 × 10^−5^; odds ratio [OR] = 3.44, 95% confidence interval [95% CI] = 1.95–6.07). Stepwise conditional analysis identified HLA-DRB1*04:05, HLA-B*39:01, and HLA-B*15:01 as being associated with independent MS susceptibility (*P*_Conditional_ < 8.3 × 10^−4^). With respect to amino acid polymorphisms in HLA genes, we found that phenylalanine at HLA-DQβ1 position 9 had the strongest effect on MS susceptibility (*P* = 3.7 × 10^−8^, OR = 3.48, 95% CI = 2.23–5.43). MS risk at HLA-DQβ1 Phe9 was independent of HLA-DRB1*15:01 (*P*_Conditional_ = 1.5 × 10^−5^, OR = 2.91, 95% CI = 1.79–4.72), while HLA-DRB1*15:01 was just significant when conditioned on HLA-DQβ1 Phe9 (*P*_Conditional_ = 0.037). Regarding a case-control analysis for NMOSD, HLA-DQA1*05:03 had a significant association with NMOSD (*P* = 1.5 × 10^−4^, OR = 6.96, 95% CI = 2.55–19.0).

**Conclusions:**

We identified HLA variants associated with the risk of MS and NMOSD. Our study contributes to the understanding of the genetic architecture of MS and NMOSD in the Japanese population.

**Electronic supplementary material:**

The online version of this article (10.1186/s12974-019-1551-z) contains supplementary material, which is available to authorized users.

## Background

The major histocompatibility complex (MHC) region at 6p21.3 is associated with a wide range of complex human diseases and quantitative traits [1–3]. In autoimmune diseases, such as rheumatoid arthritis, psoriasis, and type 1 diabetes, fine-mapping studies have identified strong genetic risk variants in the MHC region [4–6].

Multiple sclerosis (MS) is an immune-mediated demyelinating disease of the central nervous system, which affects approximately 2.3 million people worldwide [7]. Genome-wide association studies (GWAS) have reported that the MHC region is the most significant locus associated with MS [8–12]. In Europeans, previous studies have reported a relationship between classical HLA genes and MS, and found that HLA-DRB1*15:01 had the strongest effect on MS susceptibility [13]. In 2013, Patsopoulos, et al. reported that both class II HLA genes and class I HLA genes independently confer susceptibility to MS [14].

These researchers used an HLA imputation method, which computationally imputed HLA variants from single nucleotide polymorphisms (SNP) genotype data obtained from GWAS. They analyzed MS associations with eight classical HLA genes and found HLA-A*02:01 had an independent protective effect on MS. In 2018, high-resolution HLA analysis of MS by using next-generation sequencing (NGS) was conducted in Europeans [15]. The researchers found that specific class I HLA polymorphisms, such as HLA-A*02:01, HLA-C*03:04, and HLA-B*40:01 were protective for MS, independently from HLA-DRB1*15:01. In a Japanese population, Yoshimura et al. reported in 2012 that HLA-DRB1*04:05 and HLA-DPB1*03:01 were significantly associated with MS susceptibility [16]. The authors also reported that MS patients with HLA-DRB1*04:05 have a younger onset of disease. In 2016, Nakamura et al. conducted HLA analysis and found that HLA-DRB1*04:05 and HLA-DRB1*15:01 were risk factors for MS in Japanese individuals [17]. They also found that living at higher latitude and HLA-DRB1*04:05 independently affect the severity of MS.

Neuromyelitis optica spectrum disorder (NMOSD) is an immune-mediated disorder of the central nervous system that mainly affects the optic nerve and spinal cord [18]. The MHC region also contributes to the genetic architecture of NMOSD like MS [18, 19]. In Europeans, HLA-DRB1*03:01 has been reported to be significantly associated with NMOSD from a whole-genome sequencing study [19]. In the Japanese population, HLA-DRB1*16:02 and HLA-DPB1*05:01 have been reported to be positively associated with NMOSD susceptibility, and HLA-DRB1*09:01 was negatively associated with NMOSD susceptibility [20]. These Japanese studies used sequence-specific oligonucleotide hybridization [21], and only two classical HLA genes (*HLA-DRB1*, *HLA-DPB1*) were genotyped [16, 20, 21]. Thus, the classical and non-classical HLA genes affecting MS and NMOSD risk in the Japanese population have not been studied in detail. In this research, we explored the MHC region comprehensively in the Japanese population. We utilized NGS-based HLA genotyping for fine-mapping of risk [22–24] for 45 MS patients, 31 NMOSD patients, and 429 controls.

## Methods

### Sample collection

We enrolled 45 MS patients and 31 NMOSD patients. MS and NMOSD cases were collected from three medical institutes in Japan (Osaka General Medical Center, Kindai University Hospital, and Osaka University Hospital) and assessed in a retrospective manner. All subjects were of Japanese origin and provided written informed consent that was approved by the ethics committee of each hospital. All participants lived in the Kinki region which is located in the central part of Honshu Island, Japan. The Kinki region is located at latitude 35° north and longitude 135° east. MS was diagnosed by the McDonald 2010 criteria for the diagnosis of MS [25]. NMOSD was diagnosed using the international consensus diagnostic tests for NMOSD [26]. We collected clinical data of patients including sex, age, disease onset, disease duration, MS subtype, Kurtzke’s Expanded Disability Status Scale (EDSS), and anti-AQP4 antibody (Table 1). Case genomic DNA was obtained from peripheral blood. We extracted DNA using QIAGEN’s QIAampBlood Midi (Mini) Kit. This is one of the integrated DNA extraction manuals recommended by the HLA typing Quality Workshop of the Japanese Society for Histocompatibility and Immunogenetics. We used HLA genotyping data of 429 healthy individuals which were used in a previous HLA study from the Japan Biological Informatics Consortium (JBIC), as described elsewhere [6, 27]. Control genomic DNA was obtained from Epstein-Barr virus-transformed B-lymphoblast cell lines of unrelated individuals. Control genomic DNA was extracted using phenol-chloroform extraction method. All samples were checked to ensure the quality was sufficient for DNA sequencing. (OD_260nm_/OD_280nm_ = 1.8 to 2.0). We received DNA samples from JBIC.

**Table 1:** Clinical features of multiple sclerosis and neuromyelitis optica spectrum disorder patients

	MS (*n* = 45)	NMOSD (*n* = 31)
Sex (male/female)	10/35	7/24
Age (years)	43.6 (20–65)	52.7 (17–82)
Age at onset (years)	34.3 (5–58)	47.4 (15–81)
Disease duration (years)	9.3 (1–38)	5.3 (0–18)
EDSS	3.1 (0–8)	4.6 (0–9)
IgG index	0.73 (*n* = 34)	0.57 (*n* = 13)
Oligo clonal band	21/38	–
Anti-AQP4 antibody	–	29/31

This study was approved by the ethical committee of Osaka University Graduate School of Medicine.

### NGS-based HLA genotyping of classical and non-classical HLA genes

We conducted HLA genotyping (4-digit) of the 16 HLA genes in the subjects, 9 of which were classical HLA genes (class I: *HLA-A, HLA-B*, and *HLA-C*, class II: *HLA-DRA, HLA-DRB1, HLA-DQA1, HLA-DQB1, HLA-DPA1*, and *HLA-DPB1*), seven were non-classical HLA genes (*HLA-E, HLA-F, HLA-G, HLA-DOA, HLA-DOB, HLA-DMA*, and *HLA-DMB)*. HLA gene sequencing was carried out using sequence capture technology based upon hybridization between DNA of an adapter-ligated library (KAPA Hyper Prep Kit, Roche, CA, USA) and a biotinylated DNA probe (SepCap EZ choice kit, Roche, CA, USA), designed based upon target sequences of the genes in the MHC region [24]. Paired-end sequence reads were obtained using a MiSeq DNA sequencer (Illumina, the USA). Typing of 4-digit HLA alleles was conducted using Omixon Target Software (Omixon, Hungary) using the IPD-IMGT/HLA Database.

In order to supplement the HLA allele information, which was specific to the Japanese population, and was not correctly implemented in the Omixon, we updated the HLA typing results based on those obtained from a sequence-based typing method. We downloaded the GRCh37 reference genome from Ensembl, a genome browser for vertebrate genomes. GRCh37 reference genome was created by the Genome Reference Consortium (GRC) in 2009. The sequence reads were aligned to the GRCh37 of the MHC region using BWA (version 0.7.15) [28]. We obtained HLA allele sequences from the IPD and IMGT/HLA databases [29]. We used the GATK Unified Genotyper and Haplotypecaller for variant calling [30]. Details of the HLA genotyping protocol is described in our previous study [31, 32].

### The statistical framework for analysis

We evaluated the association of the HLA variants with the risk of MS and NMOSD using a logistic regression model, which assumed additive effects of allele dosages on a log-odds scale using the R statistical software (version 3.4.1) when the number of cases was more than five. Otherwise, we used Fisher’s exact test. We analyzed HLA variants that were more than 0.1% both in case and control samples. We excluded HLA amino acid polymorphisms that were monomorphic in cases or controls. We assessed the significance level of the association study by applying a Bonferroni correction according to the number of the assessed variants (adjusted *p* < 0.05). We conducted conditional association analysis of the HLA variants by including the top-associated HLA variants as covariates. An omnibus *p* value was calculated for each HLA amino acid position which had more than two amino acid residues based on a log-likelihood ratio test. We assessed the significance of the improvement in the fit by calculating the deviance for the positions with *n* residues, which followed a *χ*^*2*^ distribution with *n*-1 degrees of freedom.

### HLA amino acid 3D-structure models

We modeled the 3D-structure of HLA molecules and amino acids using the UCSF Chimera software, which is widely used for the visualization of molecular structures of proteins and three-dimensional ribbon models [33]. We downloaded the protein structure of HLA-DQ molecules from the Protein Data Bank in Europe entires 1jk8.

## Results

### NGS-based genotyping of the HLA genes in the Japanese MS and NMOSD patients and controls

For the 45 MS patients, 31 NMOSD patients, and 429 healthy controls, we conducted NGS-based genotyping of 16 HLA genes with 4-digit-level allele resolution (Additional file 1: Table S1, Table S2). Among the 16 sequenced HLA genes, 9 were classical HLA genes (class I: *HLA-A, HLA-B*, and *HLA-C*, class II: *HLA-DRA, HLA-DRB1, HLA-DQA1, HLA-DQB1, HLA-DPA1*, and *HLA-DPB1*) and 7 were non-classical HLA genes (*HLA-E, HLA-F, HLA-G, HLA-DOA, HLA-DOB, HLA-DMA,* and *HLA-DMB)*. We used a target capture technique and sequencing with relatively long read lengths to get high accuracy, as described in our previous study [27].

### Associations of HLA alleles with MS susceptibility

We evaluated the associations of the 4-digit HLA alleles with MS risk and found the most significant association to be with HLA-DRB1*15:01 (*P* = 2.1 × 10^−5^, odds ratio [OR] = 3.44, 95% confidence interval [95% CI] = 1.95–6.07; Table 2). HLA-DQB1*06:02, which was in linkage disequilibrium with HLA-DRB1*15:01 (*r*^*2*^ = 0.94 in the controls), was also significantly associated with MS (*P* = 3.0 × 10^−5^, OR = 3.45, 95% CI = 1.93–6.17; Table 2). We found that HLA-B*15:01 was significantly associated with MS (*P* = 2.2 × 10^−4^, OR = 2.95, 95% CI = 1.66–5.24; Table 2). Previous studies in Japanese and European individuals reported the strongest association of HLA-DRB1*15:01 with MS [13, 17], and our study replicated these results.

**Table 2:** HLA association analysis of multiple sclerosis in the Japanese population

Variant	Frequency		Nominal analysis		
HLA gene	MS (*n* = 45)	Control (*n* = 429)	OR (95% CI)	*P*	Fisher
HLA-B*15:01	0.200	0.078	2.95 (1.66–5.24)	2.2 × 10^−4^	
HLA-B*39:01	0.122	0.043	3.09 (1.52–6.29)	0.0019	
HLA-B*52:01	0.044	0.127	0.32 (0.08–0.88)	0.017	*
HLA-C*07:02	0.233	0.145	1.80 (1.07–3.04)	0.028	
HLA-C*12:02	0.044	0.127	0.32 (0.08–0.88)	0.017	*
HLA-DMB*01:07	0.044	0.008	5.64 (1.19–22.7)	0.015	*
HLA-DOA*01:01	0.978	0.997	0.15 (0.03–0.94)	0.042	
HLA-DPB1*05:01	0.522	0.356	1.98 (1.28–3.07)	0.0021	
HLA-DQA1*01:02	0.267	0.156	1.96 (1.19–3.25)	0.0084	
HLA-DQA1*03:03	0.222	0.141	1.74 (1.02–2.96)	0.042	
HLA-DQB1*03:01	0.033	0.104	0.30 (0.06–0.93)	0.037	*
HLA-DQB1*04:01	0.222	0.100	2.56 (1.49–4.42)	7.0 × 10^−4^	
HLA-DQB1*06:02	0.200	0.068	3.45 (1.93–6.17)	3.0 × 10^−5^	
HLA-DRA*01:01	0.733	0.579	2.00 (1.23–3.25)	0.0053	
HLA-DRA*01:02	0.267	0.421	0.50 (0.31–0.81)	0.0053	
HLA-DRB1*04:05	0.233	0.120	2.23 (1.31–3.79)	0.0030	
HLA-DRB1*15:01	0.211	0.072	3.44 (1.95–6.07)	2.1 × 10^−5^	
HLA-DRB1*15:02	0.033	0.120	0.25 (0.05–0.79)	0.012	*

*Fisher’s exact test was used for association analysis, otherwise logistic regression was used

We then conducted stepwise conditional analysis with respect to the top-associated HLA alleles. Conditional analysis of HLA-DRB1*15:01 revealed an independent association with HLA-DRB1*04:05 (*P* = 2.2 × 10^−4^, OR = 2.83, 95% CI = 1.63–4.90; Additional file 1: Table S1). Subsequent conditional analysis regarding HLA-DRB1*15:01 and HLA-DRB1*04:05 revealed an independent association with HLA-B*39:01 (*P* = 8.3 × 10^−4^, OR = 3.51, 95% CI = 1.68–7.34; Additional file 1: Table S1). Conditional analysis of HLA-DRB1*15:01, HLA-DRB1*04:05, and HLA-B*39:01 revealed an independent association with HLA-B*15:01 (*P* = 2.3 × 10^−4^, OR = 3.10, 95% CI = 1.70–5.65; Additional file 1: Table S1). After conditioning HLA-DRB1*15:01, HLA-DRB1*04:05, HLA-B*39:01, and HLA-B*15:01, no significant association locus was observed (Additional file 1: Table S1).

We then conducted a multivariate regression analysis incorporating the four associated HLA alleles (HLA-DRB1*15:01, HLA-DRB1*04:05, HLA-B*39:01, and HLA-B*15:01) and found that all the HLA alleles were independently associated with MS (HLA-DRB1*15:01 for *P* = 5.7 × 10^−6^ and OR = 4.06, HLA-DRB1*04:05 for *P* = 1.2 × 10^−4^ and OR = 3.02, and HLA-B*39:01 for *P* = 1.9 × 10^−4^ and OR = 4.13, and HLA-B*15:01 for *P* = 2.3 × 10^−4^ and OR = 3.10; Table 3). Multivariate analysis confirmed that the *HLA-B* alleles were associated with MS susceptibility independently of the *HLA-DRB1* alleles. As reported in a previous European study [14], we identified independent contributions of the class I HLA alleles to MS risk in Japanese subjects.

**Table 3:** HLA conditional analysis of multiple sclerosis in the Japanese population

Variant	Frequency		Conditional association	
			DRB1*15:01, DRB1*04:05, B*39:01, and B*15:01	
HLA gene	MS (*n* = 45)	Control (*n* = 429)	OR (95% CI)	*P*
HLA-DRB1*15:01	0.211	0.072	4.06 (2.22–7.45)	5.7 × 10^−6^
HLA-DRB1*04:05	0.233	0.120	3.02 (1.72–5.31)	1.2 × 10^−4^
HLA-B*39:01	0.122	0.043	4.13 (1.96–8.71)	1.9 × 10^− 4^
HLA-B*15:01	0.200	0.078	3.10 (1.70–5.65)	2.3 × 10^−4^

### HLA alleles with MS susceptibility and MS clinical phenotype

We identified four HLA alleles associated with MS susceptibility. We then focused on whether these HLA alleles (HLA-DRB1*15:01, HLA-DQB1*06:02, HLA-B*15:01, and HLA-B*39:01) influence the clinical course of MS. We checked the clinical feature of HLA alleles associated with MS in our research. We focused on the onset of age and EDSS score, that is widely used for disease severity and measured in all participants in this study. The results showed that no HLA alleles have a significant association with EDSS score (*P* > 0.09; Table 4A) and the onset of age (*P* > 0.14; Table 4B).

**Table 4:** HLA association analysis with the clinical course of MS

	Number of patients	EDSS	*P* (Student’s *t*)
A			
HLA-DRB1*15:01 positive	18	3.08	0.990
HLA-DRB1*15:01 negative	27	3.09	
HLA-DQB1*06:02 positive	17	3.41	0.472
HLA-DQB1*06:02 negative	28	2.89	
HLA-B*15:01 positive	15	4.20	0.098
HLA-B*15:01 negative	30	2.53	
HLA-B*39:01 positive	11	3.50	0.504
HLA-B*39:01 negative	34	2.96	
	Number of patients	Age of onset	*P* (Student’s *t*)
B			
HLA-DRB1*15:01 positive	18	33.3	0.618
HLA-DRB1*15:01 negative	27	35.0	
HLA-DQB1*06:02 positive	17	33.0	0.545
HLA-DQB1*06:02 negative	28	35.1	
HLA-B*15:01 positive	15	35.1	0.752
HLA-B*15:01 negative	30	33.9	
HLA-B*39:01 positive	11	30.0	0.142
HLA-B*39:01 negative	34	35.7	

*Student’s *t* test was used for association analysis

### Association of HLA amino acid polymorphisms with MS susceptibility

**Fig. 1:**
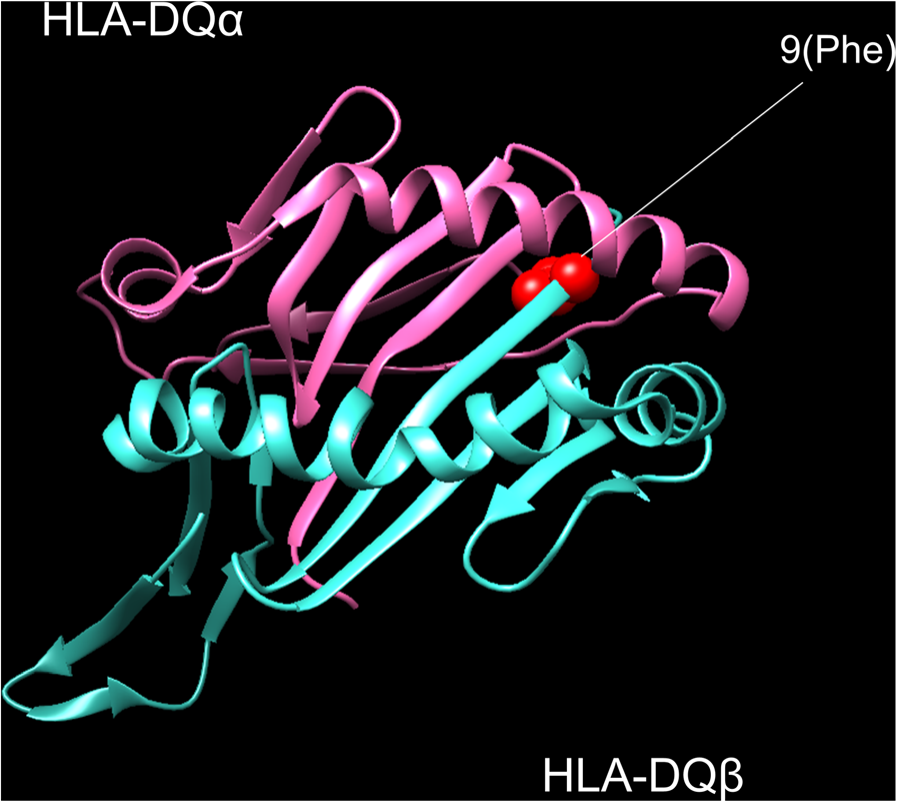
Multiple sclerosis risk-associated amino acid positions of the HLA genes in three-dimensional structure models. HLA amino acid positions with significant MS risk in HLA-DQ molecules. The protein structure is based on Protein Data Bank (PDB) entries 1jk8 and prepared using UCSF Chimera (version 1.7). Residues at the amino acid positions with significant MS risk is highlighted in red

**Fig. 2:**
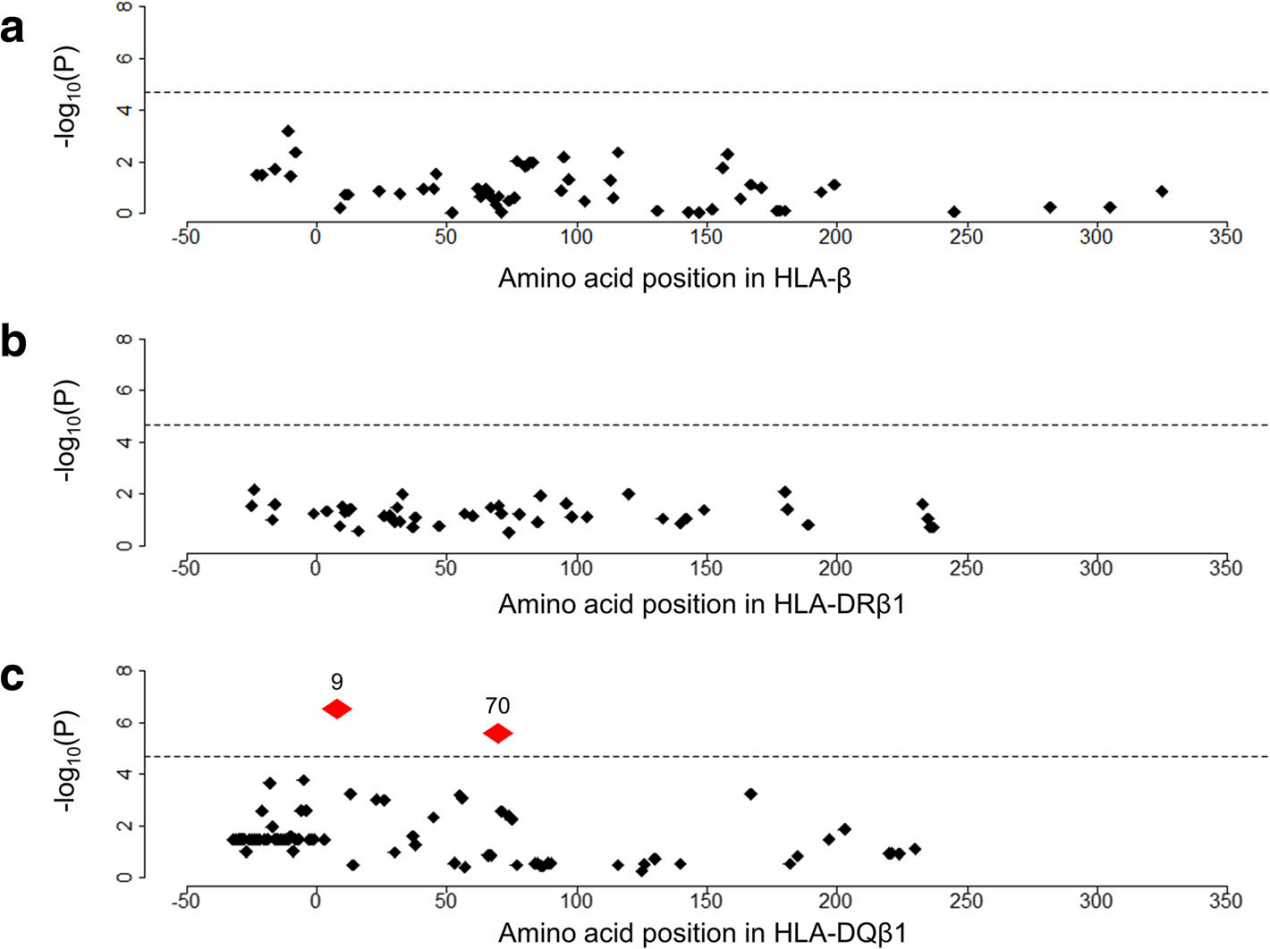
Multiple sclerosis risk-associated amino acid positions of the HLA genes. Diamonds represent the –log_10_(*P*) of the amino acid positions of the tested HLA genes. Labeled red diamonds represent the –log_10_(*P*) values of the amino acid positions significantly associated with MS. The horizontal lines represent the association *P* value of HLA-DRB1*15:01. **a** No amino acid polymorphisms of *HLA-B* was significantly associated with MS. **b** No amino acid polymorphisms of *HLA-DRB1* is significantly associated with MS. **c** The HLA-DQβ1 position 9 and 70 were associated with MS more significantly than HLA-DRB1*15:01

We then analyzed associations of the HLA amino acid polymorphisms with respect to MS susceptibility. We selected *HLA-DRB1*, *HLA-DQB1*, and *HLA-B*, which were associated with MS in our HLA allele-based study, for analysis (Additional file 1: Table S3–1). We found that the leading risk variant was the presence of phenylalanine at the HLA-DQβ1 position 9 (HLA-DQβ1 Phe9; *P* = 3.7 × 10^− 8^, OR = 3.48, 95%CI = 2.23–5.43; Additional file 1: Table S3–1, Fig. 1). We also found significant associations with the presence of arginine at HLA-DQβ1 position 70 (*P* = 2.7 × 10^−6^, OR = 0.34, 95% CI = 0.22–0.54; Additional file 1: Table S3–1), leucine at HLA-DQβ1 position − 5 (*P* = 8.9 × 10^−6^; OR = 3.54, 95% CI = 2.03–6.20; Additional file 1: Table S3–1), and valine at HLA-DQβ1 position − 18 (*P* = 2.1 × 10^−4^, OR = 2.80, 95% CI = 1.63–4.84; Additional file 1: Table S3–1). We assessed the significance of each amino acid position at HLA-DQβ1 using stepwise conditional analysis. After conditioning HLA-DQβ1 Phe9, no amino acid at HLA-DQβ1 was nominally associated with MS (Additional file 1: Table S3–2). We then conducted an omnibus test for each HLA amino acid position that had polymorphisms based on a log-likelihood ratio test [31, 32]. The HLA-DQβ1 position 9 showed the strongest association with MS (*P* = 3.6 × 10^−7^; Fig. 2), although its association was less significant than that of the binary test of HLA-DQβ1 Phe9.

### HLA-DQβ1 Phe9 independently confers MS risk with HLA-DRB1*15:01

Our study identified a significant MS risk associated with HLA-DRB1*15:01 and phenylalanine 9 at HLA-DQβ1, but it was not clear which of the variants caused these effects. We therefore conducted an analysis to investigate these effects. When conditioning HLA-DRB1*15:01, HLA-DQβ1 Phe9 was significantly associated with MS susceptibility (*P* = 1.5 × 10^−5^, OR = 2.91, 95% CI = 1.79–4.72; Additional file 1: Table S3–2). Conversely, when conditioning HLA-DQβ1 Phe9, HLA-DRB1*15:01 was just nominally significant (*P* = 0.037, OR = 1.94, 95% CI = 1.04–3.63). While further validation study should be warranted, our results suggested that HLA-DQβ1 Phe9 is a causal driver of HLA variants for MS risk in the Japanese population.

### Associations of the HLA alleles with NMOSD susceptibility

We assessed the risk of NMOSD with 4-digit HLA alleles and found the most significant association to be at HLA-DQA1*05:03 (*P* = 1.5 × 10^−4^, OR = 6.96, 95% CI = 2.55–19.0; Table 5). We conducted conditional analysis controlling for the top HLA alleles associated with NMOSD. After conditioning for HLA-DQA1*05:03, no significant association was observed after Bonferroni correction, based on the number of alleles tested (*P*_corr_ > 0.05; Additional file 1: Table S2). Our results indicated that HLA-DQA1*05:03 is a newly identified HLA allele associated with NMOSD in the Japanese population.

**Table 5:** HLA association analysis of neuromyelitis optica spectrum disorder in the Japanese population

Variant	Frequency		Nominal analysis		
HLA gene	NMOSD (*n* = 31)	Control (*n* = 429)	OR (95% CI)	*P*	Fisher
HLA-A*26:03	0.065	0.020	3.40 (0.81–10.9)	0.047	*
HLA-B*07:02	0.129	0.059	2.34 (1.06–5.19)	0.036	
HLA-DPB1*05:01	0.516	0.356	1.93 (1.15–3.24)	0.012	
HLA-DQA1*01:01	0.129	0.058	2.39 (1.08–5.30)	0.031	
HLA-DQA1*03:02	0.048	0.147	0.30 (0.06–0.93)	0.035	*
HLA-DQA1*05:03	0.097	0.015	6.96 (2.55–19.0)	1.5 × 10^−4^	
HLA-DQB1*03:01	0.210	0.104	2.29 (1.20–4.39)	0.012	
HLA-DQB1*03:03	0.048	0.149	0.29 (0.06–0.91)	0.024	*
HLA-DRB1*01:01	0.129	0.058	2.39 (1.08–5.30)	0.031	
HLA-DRB1*09:01	0.016	0.135	0.10 (0.003–0.62)	0.0027	*
HLA-DRB1*14:06	0.081	0.012	7.40 (1.92–24.8)	0.0021	*

*Fisher’s exact test was used for association analysis, otherwise, logistic regression was used

### Associations of the HLA amino acid polymorphisms with NMOSD susceptibility

We analyzed the association of the HLA amino acid polymorphisms with NMOSD susceptibility. We selected *HLA-DQA1*, which was associated with NMOSD in our HLA allele-based association study. We found no amino acid variants with significant risk after Bonferroni correction, based on the number of amino acids tested (*P*_corr_ > 0.05; Additional file 1: Table S4).

## Discussion

In this study, we performed HLA analysis of MS/NMOSD in Japanese individuals. We replicated results indicating that HLA-DRB1*15:01 is the allele most strongly associated with MS susceptibility, a finding which was comparable with those of previous Japanese and European populations [13, 17]. HLA-B*39:01 and HLA-B*15:01 also appeared to be associated with MS. Previous European studies have reported an association of class I HLA alleles with MS susceptibility [14]. These results suggest a relationship between class I HLA alleles and MS susceptibility, as well as class II HLA alleles, in both Europeans and Japanese.

We also conducted an association study involving HLA amino acid polymorphisms and found that HLA-DQβ1 amino acid polymorphisms led to a significantly increased risk of MS. Previous studies have reported the importance of *HLA-DRB1* with respect to MS [14, 34], but the role of *HLA-DQB1* has not been studied in detail. In our study, even conditioning HLA-DRB1*15:01, HLA amino acid polymorphisms at HLA-DQβ1 were associated with increased risk of MS. This result indicates that HLA-DQβ1 polymorphisms could independently confer risk of MS. The association of HLA-DQB1 with MS has not been reported well. MHC class II molecules are heterodimers of an alpha and a beta chain, and have a peptide-binding site for presenting antigenic peptides to T cells [35]. In this study, we found that two amino acid positions associated with MS (HLA-DQβ1 position 9 and 70). Previous studies showed that HLA-DQβ1 position 9 is in the peptide-binding groove of HLA-DQ molecules and HLA-DQβ1 position 70 residues in the putative T cell receptor (TCR) [36]. Within the central nervous system, antigen-presenting cells such as microglia and astrocytes work as defenders against infections and inflammation [37]. Changes in antigen-presenting function of peptide binding amino acid changes could influence the susceptibility to MS. Animal model studies have found that susceptibility to myelin oligodendrocyte-binding protein or proteolipid protein were determined by HLA-DQB1*06:02 [38, 39]. These findings also suggest the possibility that *HLA-DQB1* plays an important role in susceptibility to MS.

HLA-DQA1*05:03 was identified as being associated with NMOSD in our study. *HLA-DQA1* has been reported to be associated with immune-mediated diseases such as celiac disease and type 1 diabetes [40, 41]. In Europeans, a recent GWAS identified rs28383224, which is located in *HLA-DQA1*, as having the strongest association with NMOSD susceptibility [19]. Although the role of the *HLA-DQA1* risk SNP has not been investigated in detail, the importance of *HLA-DQA1* in NMOSD pathophysiology was suggested.

A previous study in Japan reported that HLA-DRB1*16:02 and HLA-DPB1*05:01 presented a significantly elevated risk of NMOSD, while HLA-DRB1*09:01 reduced the risk. [22] In our analysis, HLA-DRB1*09:01 and HLA-DPB1*05:01 were nominally significant (HLA-DRB1*09:01; *P* = 0.0027, OR = 0.10, 95% CI = 0.003–0.62, HLA-DPB1*05:01; *P* = 0.012, OR = 1.93, 95% CI = 1.15–3.24; Additional file 1 Table S2), but after performing Bonferroni correction, they were not significantly associated with NMOSD. This result may be because our study sample size was smaller than that of the previous study in Japan. HLA-DRB1*16:02 was not significant in our study (*P* = 0.39, OR = 2.32, 95% CI = 0.05–19.6). These findings may indicate that the HLA alleles associated with NMOSD are localized in Japan. Further studies are needed to identify the details of the variability of HLA alleles in the Japanese population.

## Conclusions

In summary, through NGS-based HLA typing of the MS and NMOSD patients and controls of Japanese ancestry, we analyzed the variants of the 16 HLA genes. We newly identified the risk HLA alleles associated with MS and NMOSD risk. We also identified that the amino acid polymorphisms at HLA-DQβ1 are more strongly associated with MS susceptibility than HLA alleles. Our study contributes to the understanding of the genetic background of MS and NMOSD in Japanese.

## Additional file

Additional file 1: Table S1 Results of the association analysis of HLA alleles (multiple sclerosis). Table S2 Results of the association analysis of HLA alleles (neuromyelitis optica spectrum disorder). Table S3–1 Results of the HLA amino acid association analysis (multiple sclerosis). Table S3–2 Results of the conditional HLA DQβ1 association analysis (multiple sclerosis). Table S4 Results of the HLA amino acid association analysis (neuromyelitis optica spectrum disorder). (PDF 542 kb)

## Data Availability

The datasets used and analyzed during the current study are available from the corresponding author on reasonable request.

## Abbreviations

GWAS: Genome-wide association studies
MHC: Major histocompatibility complex
MS: Multiple sclerosis
NGS: Next-generation sequencing
NMOSD: Neuromyelitis optica spectrum disorder
SNP: Single-nucleotide polymorphisms
