# Supplementary material for: Next-generation sequencing identifies contribution of both class I and II HLA genes on susceptibility of multiple sclerosis in Japanese
Source: J Neuroinflammation. 2019 Aug 5;16:162. doi: 10.1186/s12974-019-1551-z (PMC6683481; doi:10.1186/s12974-019-1551-z)
Supplement: Supplementary file 1 — Table S1 Results of the association analysis of HLA alleles (multiple sclerosis). Table S2 Results of the association analysis of HLA alleles (neuromyelitis optica spectrum disorder). Table S3–1 Results of the HLA amino acid association analysis (multiple sclerosis). Table S3–2 Results of the conditional HLA DQβ1 association analysis (multiple sclerosis). Table S4 Results of the HLA amino acid association analysis (neuromyelitis optica spectrum disorder). (PDF 542 kb) [file 12974_2019_1551_MOESM1_ESM.pdf]

Table S1. Results of the association analysis of HLA alleles (multiple sclerosis)

						Conditional association							
						DRB1*15:01		DRB1*15:01, and DRB1*04:05		DRB1*15:01, DRB1*04:05, and B*39:01		DRB1*15:01, DRB1*04:05, B*39:01, and B*15:01	
Variant	Frequency												
(112 genes)	MS (n=45)	Control (n=429)	OR (95%CI)	P	Fisher	OR (95%CI)	P	OR (95%CI)	P	OR (95%CI)	P	OR (95%CI)	P
HLA-A													
HLA-A*02:01	0.144	0.107	1.41 (0.75-2.63)	0.29		1.44 (0.77-2.72)	0.26	1.36 (0.72-2.58)	0.35	1.25 (0.65-2.40)	0.50	1.22 (0.64-2.35)	0.55
HLA-A*02:06	0.067	0.087	0.75 (0.32-1.76)	0.50		0.76 (0.32-1.81)	0.53	0.66 (0.27-1.58)	0.35	0.56 (0.23-1.36)	0.20	0.52 (0.21-1.28)	0.16
HLA-A*02:07	0.022	0.023	0.95 (0.11-4.03)	1.0	*	—	—	—	—	—	—	—	—
HLA-A*11:01	0.144	0.103	1.48 (0.79-2.77)	0.22		1.51 (0.80-2.85)	0.20	1.29 (0.68-2.47)	0.44	1.23 (0.64-2.36)	0.54	1.05 (0.54-2.05)	0.88
HLA-A*24:02	0.322	0.386	0.76 (0.48-1.20)	0.24		0.76 (0.48-1.22)	0.26	0.80 (0.50-1.28)	0.35	0.85 (0.53-1.38)	0.51	0.89 (0.55-1.43)	0.62
HLA-A*24:20	0.011	0.008	1.37 (0.03-10.8)	0.55	*	—	—	—	—	—	—	—	—
HLA-A*26:01	0.078	0.078	1.00 (0.44-2.24)	0.99		0.93 (0.41-2.11)	0.86	0.98 (0.43-2.25)	0.97	1.02 (0.44-2.34)	0.97	0.98 (0.42-2.28)	0.96
HLA-A*26:02	0.011	0.022	0.50 (0.01-3.20)	0.71	*	—	—	—	—	—	—	—	—
HLA-A*26:03	0.044	0.020	2.30 (0.55-7.26)	0.13	*	—	—	—	—	—	—	—	—
HLA-A*31:01	0.078	0.065	1.21 (0.53-2.74)	0.65		1.12 (0.49-2.58)	0.79	1.19 (0.52-2.76)	0.68	1.29 (0.56-2.99)	0.56	1.56 (0.66-3.65)	0.31
HLA-A*33:03	0.067	0.078	0.84 (0.36-2.00)	0.70		0.92 (0.39-2.20)	0.85	1.02 (0.42-2.45)	0.97	1.13 (0.47-2.75)	0.78	1.36 (0.55-3.32)	0.50
HLA-B													
HLA-B*07:02	0.067	0.059	1.13 (0.47-2.71)	0.78		1.35 (0.56-3.26)	0.51	1.52 (0.62-3.71)	0.36	1.71 (0.69-4.19)	0.25	2.03 (0.82-5.04)	0.13
HLA-B*15:01	0.200	0.078	2.95 (1.66-5.24)	2.2×10^{-4}		2.71 (1.51-4.87)	8.4×10^{-4}	2.73 (1.51-4.93)	8.9×10^{-4}	3.10 (1.70-5.65)	2.3×10^{-4}	—	—
HLA-B*15:11	0.022	0.003	6.45 (0.53-57.1)	0.074	*	—	—	—	—	—	—	—	—
HLA-B*35:01	0.044	0.059	0.74 (0.19-2.08)	0.81	*	—	—	—	—	—	—	—	—
HLA-B*39:01	0.122	0.043	3.09 (1.52-6.29)	0.0019		3.20 (1.55-6.60)	0.0016	3.51 (1.68-7.34)	8.3×10^{-4}	—	—	—	—
HLA-B*40:01	0.067	0.059	1.13 (0.47-2.71)	0.78		1.17 (0.48-2.83)	0.73	1.06 (0.43-2.59)	0.90	1.17 (0.47-2.86)	0.74	1.39 (0.56-3.45)	0.47
HLA-B*40:02	0.056	0.077	0.71 (0.22-1.80)	0.67	*	—	—	—	—	—	—	—	—
HLA-B*40:03	0.011	0.002	4.79 (0.08-93.1)	0.26	*	—	—	—	—	—	—	—	—
HLA-B*40:06	0.044	0.056	0.79 (0.20-2.22)	0.81	*	—	—	—	—	—	—	—	—
HLA-B*44:03	0.033	0.076	0.42 (0.08-1.33)	0.19	*	—	—	—	—	—	—	—	—
HLA-B*46:01	0.022	0.047	0.47 (0.05-1.85)	0.42	*	—	—	—	—	—	—	—	—
HLA-B*48:01	0.033	0.037	0.89 (0.17-2.94)	1.0	*	—	—	—	—	—	—	—	—
HLA-B*51:01	0.089	0.075	1.21 (0.56-2.61)	0.63		0.97 (0.44-2.15)	0.94	0.99 (0.44-2.20)	0.98	1.10 (0.49-2.45)	0.83	1.36 (0.60-3.07)	0.46
HLA-B*52:01	0.044	0.127	0.32 (0.08-0.88)	0.017	*	—	—	—	—	—	—	—	—
HLA-B*54:01	0.067	0.069	0.97 (0.41-2.31)	0.94		0.82 (0.34-2.00)	0.67	0.82 (0.34-2.00)	0.66	0.90 (0.37-2.21)	0.82	1.10 (0.44-2.72)	0.84
HLA-B*55:02	0.011	0.020	0.56 (0.01-3.63)	1.0	*	—	—	—	—	—	—	—	—
HLA-B*58:01	0.022	0.005	4.84 (0.43-34.3)	0.10	*	—	—	—	—	—	—	—	—
HLA-B*59:01	0.011	0.016	0.68 (0.02-4.55)	1.0	*	—	—	—	—	—	—	—	—
HLA-B*67:01	0.033	0.012	2.92 (0.51-11.6)	0.12	*	—	—	—	—	—	—	—	—
HLA-C													
HLA-C*01:02	0.122	0.156	0.75 (0.39-1.45)	0.40		0.82 (0.42-1.60)	0.57	0.57 (0.28-1.16)	0.12	0.57 (0.28-1.16)	0.12	0.59 (0.29-1.20)	0.15
HLA-C*01:03	0.011	0.002	4.79 (0.08-93.1)	0.26	*	—	—	—	—	—	—	—	—
HLA-C*03:02	0.022	0.005	4.84 (0.43-34.3)	0.10	*	—	—	—	—	—	—	—	—
HLA-C*03:03	0.167	0.111	1.61 (0.89-2.91)	0.12		1.58 (0.87-2.88)	0.14	1.45 (0.79-2.67)	0.23	1.59 (0.86-2.94)	0.14	1.17 (0.61-2.27)	0.64
HLA-C*03:04	0.111	0.128	0.85 (0.43-1.69)	0.64		0.87 (0.43-1.73)	0.68	0.85 (0.42-1.71)	0.66	0.79 (0.39-1.60)	0.51	0.85 (0.41-1.73)	0.65
HLA-C*04:01	0.067	0.041	1.68 (0.69-4.11)	0.26		1.74 (0.70-4.31)	0.23	1.91 (0.76-4.77)	0.17	1.95 (0.78-4.90)	0.16	1.19 (0.44-3.21)	0.73
HLA-C*07:02	0.233	0.145	1.80 (1.07-3.04)	0.028		1.76 (1.04-3.00)	0.037	1.98 (1.15-3.41)	0.014	1.65 (0.93-2.92)	0.087	1.66 (0.93-2.94)	0.087
HLA-C*08:01	0.089	0.079	1.13 (0.53-2.44)	0.75		1.12 (0.52-2.44)	0.77	1.14 (0.52-2.49)	0.74	1.18 (0.53-2.61)	0.68	1.33 (0.60-2.96)	0.49
HLA-C*08:03	0.022	0.015	1.48 (0.16-6.69)	0.65	*	—	—	—	—	—	—	—	—
HLA-C*12:02	0.044	0.127	0.32 (0.08-0.88)	0.017	*	—	—	—	—	—	—	—	—
HLA-C*14:02	0.078	0.061	1.31 (0.58-2.97)	0.52		1.10 (0.47-2.55)	0.83	1.22 (0.52-2.86)	0.65	1.36 (0.58-3.21)	0.49	1.68 (0.70-4.00)	0.24
HLA-C*14:03	0.033	0.076	0.42 (0.08-1.33)	0.19	*	—	—	—	—	—	—	—	—
HLA-DMA													
HLA-DMA*01:01	0.811	0.852	0.75 (0.43-1.31)	0.31		0.73 (0.41-1.28)	0.27	0.72 (0.40-1.27)	0.25	0.73 (0.41-1.31)	0.29	0.67 (0.37-1.21)	0.19
HLA-DMA*01:02	0.189	0.148	1.34 (0.77-2.35)	0.31		1.38 (0.78-2.43)	0.27	1.40 (0.79-2.48)	0.25	1.36 (0.76-2.44)	0.29	1.49 (0.83-2.69)	0.19
HLA-DMB													
HLA-DMB*01:01	0.422	0.493	0.75 (0.48-1.17)	0.20		0.89 (0.56-1.40)	0.61	0.81 (0.51-1.29)	0.38	0.76 (0.47-1.21)	0.25	0.71 (0.44-1.14)	0.16
HLA-DMB*01:02	0.211	0.191	1.13 (0.66-1.93)	0.65		1.13 (0.66-1.94)	0.66	1.17 (0.68-2.02)	0.57	1.24 (0.71-2.14)	0.45	1.12 (0.64-1.95)	0.70
HLA-DMB*01:03	0.322	0.307	1.08 (0.68-1.71)	0.76		0.87 (0.54-1.42)	0.58	0.96 (0.58-1.57)	0.86	0.98 (0.59-1.62)	0.93	1.13 (0.68-1.88)	0.64
HLA-DMB*01:07	0.044	0.008	5.64 (1.19-22.7)	0.015	*	—	—	—	—	—	—	—	—
HLA-DOA													
HLA-DOA*01:01	0.978	0.997	0.15 (0.03-0.94)	0.042		0.13 (0.02-0.79)	0.027	0.10 (0.02-0.64)	0.015	0.12 (0.02-0.78)	0.027	0.11 (0.02-0.70)	0.020
HLA-DOA*01:04	0.022	0.003	6.45 (0.53-57.1)	0.074	*	—	—	—	—	—	—	—	—
HLA-DOB													
HLA-DOB*01:01	0.800	0.768	1.21 (0.70-2.07)	0.49		1.69 (0.94-3.04)	0.077	1.58 (0.87-2.85)	0.13	1.53 (0.84-2.78)	0.16	1.44 (0.79-2.61)	0.24
HLA-DOB*01:02	0.156	0.182	0.83 (0.46-1.50)	0.54		0.54 (0.28-1.05)	0.068	0.57 (0.29-1.10)	0.093	0.58 (0.30-1.12)	0.10	0.60 (0.31-1.15)	0.13
HLA-DOB*01:04	0.044	0.050	0.88 (0.22-2.51)	1.0	*	—	—	—	—	—	—	—	—
HLA-DPA1													
HLA-DPA1*01:03	0.367	0.406	0.85 (0.54-1.33)	0.47		0.93 (0.59-1.48)	0.77	0.88 (0.55-1.39)	0.57	0.79 (0.49-1.27)	0.34	0.72 (0.45-1.17)	0.18
HLA-DPA1*02:01	0.144	0.171	0.82 (0.44-1.51)	0.52		0.84 (0.45-1.57)	0.59	0.89 (0.48-1.67)	0.73	0.93 (0.50-1.74)	0.82	0.88 (0.46-1.66)	0.68
HLA-DPA1*02:02	0.478	0.423	1.25 (0.81-1.93)	0.32		1.12 (0.72-1.75)	0.61	1.16 (0.74-1.81)	0.52	1.24 (0.79-1.96)	0.35	1.41 (0.89-2.25)	0.15
HLA-DPB1													
HLA-DPB1*02:01	0.189	0.220	0.82 (0.48-1.43)	0.49		0.85 (0.48-1.48)	0.56	0.86 (0.49-1.51)	0.60	0.79 (0.45-1.40)	0.42	0.69 (0.39-1.23)	0.21
HLA-DPB1*03:01	0.089	0.055	1.68 (0.77-3.68)	0.19		1.69 (0.76-3.74)	0.20	1.47 (0.66-3.28)	0.35	1.35 (0.60-3.07)	0.47	1.22 (0.53-2.81)	0.64
HLA-DPB1*04:01	0.011	0.057	0.19 (0.01-1.11)	0.078	*	—	—	—	—	—	—	—	—
HLA-DPB1*04:02	0.078	0.093	0.82 (0.37-1.83)	0.63		0.87 (0.38-1.95)	0.73	0.81 (0.36-1.83)	0.61	0.84 (0.37-1.91)	0.67	0.90 (0.39-2.05)	0.80
HLA-DPB1*05:01	0.522	0.356	1.98 (1.28-3.07)	0.0021		1.85 (1.19-2.88)	0.0063	1.82 (1.17-2.85)	0.0083	1.93 (1.23-3.03)	0.0044	1.92 (1.22-3.03)	0.0050
HLA-DPB1*06:01	0.011	0.012	0.95 (0.02-6.84)	1.0	*	—	—	—	—	—	—	—	—
HLA-DPB1*09:01	0.067	0.118	0.54 (0.23-1.26)	0.15		0.58 (0.24-1.36)	0.21	0.66 (0.28-1.57)	0.35	0.73 (0.30-1.73)	0.47	0.83 (0.35-2.01)	0.69
HLA-DPB1*13:01	0.022	0.015	1.48 (0.16-6.69)	0.65	*	—	—	—	—	—	—	—	—
HLA-DPB1*14:01	0.011	0.014	0.79 (0.02-5.47)	1.0	*	—	—	—	—	—	—	—	—
HLA-DQA1													
HLA-DQA1*01:01	0.044	0.058	0.75 (0.19-2.12)	0.81	*	—	—	—	—	—	—	—	—
HLA-DQA1*01:02	0.267	0.156	1.96 (1.19-3.25)	0.0084		1.62 (0.96-2.73)	0.071	1.60 (0.95-2.71)	0.080	1.61 (0.95-2.75)	0.078	1.41 (0.81-2.45)	0.22
HLA-DQA1*01:03	0.122	0.204	0.54 (0.28-1.04)	0.067		0.59 (0.30-1.13)	0.11	0.59 (0.31-1.15)	0.12	0.53 (0.27-1.04)	0.065	0.52 (0.26-1.01)	0.055
HLA-DQA1*01:04	0.067	0.063	1.06 (0.44-2.55)	0.89		1.18 (0.49-2.85)	0.71	1.22 (0.50-2.97)	0.66	1.00 (0.40-2.50)	0.99	0.99 (0.39-2.47)	0.97
HLA-DQA1*01:05	0.011	0.003	3.20 (0.06-40.3)	0.33	*	—	—	—	—	—	—	—	—
HLA-DQA1*03:01	0.089	0.089	1.00 (0.47-2.15)	0.99		0.99 (0.46-2.13)	0.97	1.03 (0.47-2.25)	0.94	1.12 (0.51-2.45)	0.77	0.99 (0.45-2.19)	0.98
HLA-DQA1*03:02	0.078	0.147	0.49 (0.22-1.08)	0.078		0.51 (0.23-1.14)	0.10	0.52 (0.23-1.16)	0.11	0.53 (0.24-1.19)	0.13	0.55 (0.25-1.24)	0.15
HLA-DQA1*03:03	0.222	0.141	1.74 (1.02-2.96)	0.042		1.71 (1.00-2.94)	0.050	1.39 (0.79-2.44)	0.25	1.53 (0.86-2.70)	0.15	1.76 (0.98-3.17)	0.057
HLA-DQA1*04:01	0.067	0.034	2.04 (0.82-5.06)	0.12		2.19 (0.87-5.48)	0.10	2.28 (0.90-5.79)	0.082	2.11 (0.83-5.39)	0.12	2.25 (0.88-5.76)	0.092
HLA-DQA1*05:03	0.011	0.015	0.73 (0.02-4.97)	1.0	*	—	—	—	—	—	—	—	—
HLA-DQA1*05:05	0.011	0.043	0.25 (0.01-1.52)	0.25	*	—	—	—	—	—	—	—	—
HLA-DQA1*05:07	0.011	0.013	0.87 (0.02-6.08)	1.0	*	—	—	—	—	—	—	—	—
HLA-DQB1													
HLA-DQB1*03:01	0.033	0.104	0.30 (0.06-0.93)	0.037	*	—	—	—	—	—	—	—	—
HLA-DQB1*03:02	0.078	0.098	0.78 (0.35-1.74)	0.54		0.86 (0.38-1.94)	0.72	0.87 (0.38-1.96)	0.74	0.91 (0.40-2.06)	0.81	0.69 (0.30-1.62)	0.40
HLA-DQB1*03:03	0.089	0.149	0.56 (0.26-1.18)	0.13		0.66 (0.31-1.42)	0.29	0.71 (0.33-1.53)	0.39	0.68 (0.32-1.47)	0.33	0.65 (0.30-1.41)	0.27

HLA-DQB1*04:01	0.222	0.100	2.56 (1.49-4.42)	7.0×10^{-4}		2.80 (1.61-4.87)	2.8×10^{-4}	1.97 (1.03-3.75)	0.040	1.75 (0.91-3.39)	0.094	1.85 (0.95-3.61)	0.072
HLA-DQB1*04:02	0.067	0.048	1.42 (0.59-3.45)	0.44		1.63 (0.67-3.97)	0.29	1.53 (0.62-3.78)	0.36	1.52 (0.61-3.77)	0.37	1.52 (0.61-3.81)	0.37
HLA-DQB1*05:01	0.056	0.063	0.88 (0.27-2.26)	1.0	*	—	—	—	—	—	—	—	—
HLA-DQB1*05:02	0.022	0.028	0.79 (0.09-3.27)	1.0	*	—	—	—	—	—	—	—	—
HLA-DQB1*05:03	0.056	0.044	1.27 (0.38-3.35)	0.59	*	—	—	—	—	—	—	—	—
HLA-DQB1*06:01	0.122	0.199	0.56 (0.29-1.07)	0.081		0.62 (0.32-1.19)	0.15	0.75 (0.38-1.47)	0.40	0.78 (0.40-1.54)	0.47	0.83 (0.42-1.65)	0.59
HLA-DQB1*06:02	0.200	0.068	3.45 (1.93-6.17)	3.0×10^{-5}		2.00 (0.86-4.67)	0.11	1.96 (0.84-4.59)	0.12	1.99 (0.85-4.64)	0.11	2.22 (0.97-5.09)	0.060
HLA-DQB1*06:04	0.033	0.076	0.42 (0.08-1.33)	0.19	*	—	—	—	—	—	—	—	—
HLA-DQB1*06:09	0.022	0.003	6.45 (0.53-57.1)	0.074	*	—	—	—	—	—	—	—	—
HLA-DRA													
HLA-DRA*01:01	0.733	0.579	2.00 (1.23-3.25)	0.0053		1.85 (1.13-3.03)	0.014	1.54 (0.92-2.57)	0.10	1.47 (0.87-2.46)	0.15	1.27 (0.75-2.16)	0.37
HLA-DRA*01:02	0.267	0.421	0.50 (0.31-0.81)	0.0053		0.54 (0.33-0.88)	0.014	0.65 (0.39-1.09)	0.10	0.68 (0.41-1.15)	0.15	0.79 (0.46-1.33)	0.37
HLA-DRB1													
HLA-DRB1*01:01	0.044	0.058	0.75 (0.19-2.12)	0.81	*	—	—	—	—	—	—	—	—
HLA-DRB1*04:03	0.011	0.020	0.56 (0.01-3.63)	1.0	*	—	—	—	—	—	—	—	—
HLA-DRB1*04:05	0.233	0.120	2.23 (1.31-3.79)	0.0030		2.83 (1.63-4.90)	2.2×10^{-4}	—	—	—	—	—	—
HLA-DRB1*04:06	0.067	0.034	2.04 (0.82-5.06)	0.12		2.44 (0.98-6.09)	0.056	3.12 (1.23-7.92)	0.017	3.55 (1.39-9.07)	0.0080	2.24 (0.82-6.12)	0.12
HLA-DRB1*08:02	0.044	0.056	0.79 (0.20-2.22)	0.81	*	—	—	—	—	—	—	—	—
HLA-DRB1*08:03	0.078	0.079	0.98 (0.44-2.20)	0.96		1.17 (0.52-2.66)	0.71	1.50 (0.65-3.46)	0.35	1.25 (0.53-2.95)	0.61	1.29 (0.54-3.07)	0.56
HLA-DRB1*09:01	0.089	0.135	0.62 (0.29-1.32)	0.22		0.74 (0.35-1.59)	0.45	0.95 (0.43-2.07)	0.89	0.94 (0.43-2.07)	0.88	0.92 (0.42-2.02)	0.83
HLA-DRB1*12:01	0.033	0.030	1.10 (0.21-3.71)	0.75	*	—	—	—	—	—	—	—	—
HLA-DRB1*13:02	0.056	0.078	0.69 (0.21-1.77)	0.54	*	—	—	—	—	—	—	—	—
HLA-DRB1*14:03	0.022	0.013	1.75 (0.19-8.20)	0.35	*	—	—	—	—	—	—	—	—
HLA-DRB1*14:05	0.044	0.027	1.69 (0.41-5.10)	0.31	*	—	—	—	—	—	—	—	—
HLA-DRB1*14:54	0.022	0.036	0.61 (0.07-2.45)	0.76	*	—	—	—	—	—	—	—	—
HLA-DRB1*15:01	0.211	0.072	3.44 (1.95-6.07)	2.1×10^{-5}		—	—	—	—	—	—	—	—
HLA-DRB1*15:02	0.033	0.120	0.25 (0.05-0.79)	0.012	*	—	—	—	—	—	—	—	—
HLA-E													
HLA-E*01:01	0.344	0.307	1.19 (0.75-1.88)	0.46		1.25 (0.79-1.99)	0.35	1.13 (0.70-1.81)	0.62	1.04 (0.64-1.68)	0.88	0.92 (0.56-1.50)	0.74
HLA-E*01:03	0.656	0.694	0.84 (0.53-1.33)	0.46		0.80 (0.50-1.27)	0.35	0.89 (0.55-1.42)	0.62	0.96 (0.60-1.56)	0.88	1.09 (0.67-1.77)	0.74
HLA-F													
HLA-F*01:01	0.978	0.980	0.89 (0.20-3.91)	0.88		0.92 (0.21-4.13)	0.91	1.01 (0.22-4.59)	0.99	1.00 (0.22-4.54)	1.0	0.86 (0.19-3.90)	0.84
HLA-F*01:05	0.022	0.014	1.60 (0.17-7.37)	0.63	*	—	—	—	—	—	—	—	—
HLA-G													
HLA-G*01:01	0.578	0.511	1.31 (0.85-2.04)	0.23		1.34 (0.86-2.09)	0.20	1.22 (0.78-1.91)	0.39	1.11 (0.70-1.76)	0.65	0.90 (0.56-1.47)	0.68
HLA-G*01:03	0.011	0.001	9.58 (0.12-752)	0.18	*	—	—	—	—	—	—	—	—
HLA-G*01:04	0.411	0.479	0.76 (0.49-1.18)	0.22		0.75 (0.48-1.16)	0.20	0.82 (0.52-1.29)	0.38	0.89 (0.56-1.42)	0.63	1.09 (0.67-1.77)	0.73

* Fisher's exact test was used for association analysis, otherwise logistic regression was used.

Table S2. Results of the association analysis of HLA alleles (neuromyelitis optica spectrum disorder)

Variant	Frequency		Nominal analysis			Conditional association DQA1*05:03	
(104 genes)	NMO (n=31)	Control (n=429)	OR (95%CI)	*P*	Fisher	OR(95%CI)	*P*
HLA-A							
HLA-A*02:01	0.113	0.107	1.06 (0.47-2.40)	0.89		1.17 (0.51-2.66)	0.71
HLA-A*02:06	0.065	0.087	0.72 (0.18-2.03)	0.81	*	—	—
HLA-A*11:01	0.113	0.103	1.11 (0.49-2.52)	0.80		1.17 (0.51-2.66)	0.71
HLA-A*24:02	0.323	0.386	0.76 (0.44-1.31)	0.32		0.79 (0.45-1.37)	0.40
HLA-A*26:01	0.113	0.078	1.50 (0.66-3.43)	0.33		1.48 (0.64-3.41)	0.36
HLA-A*26:02	0.048	0.022	2.24 (0.41-7.94)	0.18	*	—	—
HLA-A*26:03	0.065	0.020	3.40 (0.81-10.9)	0.047	*	—	—
HLA-A*31:01	0.129	0.065	2.12 (0.96-4.68)	0.062		1.77 (0.78-4.05)	0.17
HLA-A*33:03	0.032	0.078	0.39 (0.05-1.55)	0.31	*	—	—
HLA-B							
HLA-B*07:02	0.129	0.059	2.34 (1.06-5.19)	0.036		2.59 (1.17-5.78)	0.020
HLA-B*15:01	0.097	0.078	1.26 (0.53-3.04)	0.60		1.24 (0.51-3.01)	0.64
HLA-B*15:07	0.016	0.003	4.66 (0.09-59.0)	0.24	*	—	—
HLA-B*35:01	0.065	0.059	1.09 (0.28-3.13)	0.78	*	—	—
HLA-B*39:01	0.081	0.043	1.94 (0.57-5.23)	0.20	*	—	—
HLA-B*40:01	0.065	0.059	1.09 (0.28-3.13)	0.78	*	—	—
HLA-B*40:02	0.113	0.077	1.53 (0.67-3.49)	0.31		1.50 (0.65-3.47)	0.34
HLA-B*44:03	0.032	0.076	0.41 (0.05-1.60)	0.31	*	—	—
HLA-B*46:01	0.048	0.047	1.04 (0.20-3.42)	1.0	*	—	—
HLA-B*48:01	0.048	0.037	1.31 (0.25-4.39)	0.73	*	—	—
HLA-B*51:01	0.081	0.075	1.09 (0.33-2.83)	0.80	*	—	—
HLA-B*52:01	0.113	0.127	0.88 (0.39-1.97)	0.75		0.92 (0.41-2.09)	0.85
HLA-B*54:01	0.048	0.069	0.69 (0.13-2.22)	0.79	*	—	—
HLA-B*55:02	0.032	0.020	1.65 (0.18-7.21)	0.37	*	—	—
HLA-B*59:01	0.032	0.016	2.01 (0.22-9.06)	0.29	*	—	—
HLA-C							
HLA-C*01:02	0.161	0.156	1.04 (0.52-2.10)	0.91		1.15 (0.57-2.34)	0.69
HLA-C*01:03	0.016	0.002	6.98 (0.12-136)	0.19	*	—	—
HLA-C*03:03	0.145	0.111	1.36 (0.65-2.85)	0.41		1.38 (0.66-2.91)	0.40
HLA-C*03:04	0.129	0.128	1.01 (0.47-2.17)	0.98		0.99 (0.45-2.15)	0.97
HLA-C*04:01	0.032	0.041	0.78 (0.09-3.18)	1.0	*	—	—
HLA-C*07:02	0.226	0.145	1.73 (0.92-3.23)	0.087		1.59 (0.84-3.01)	0.16
HLA-C*08:01	0.032	0.079	0.39 (0.05-1.52)	0.22	*	—	—
HLA-C*08:03	0.032	0.015	2.16 (0.23-9.90)	0.27	*	—	—
HLA-C*12:02	0.113	0.127	0.87 (0.39-1.97)	0.75		0.89 (0.39-2.01)	0.77
HLA-C*14:02	0.065	0.061	1.07 (0.27-3.06)	0.79	*	—	—
HLA-C*14:03	0.032	0.076	0.41 (0.05-1.60)	0.31	*	—	—
HLA-C*15:02	0.016	0.022	0.72 (0.02-4.71)	1.0	*	—	—
HLA-DMA							
HLA-DMA*01:01	0.807	0.852	0.72 (0.38-1.40)	0.34		0.99 (0.48-2.05)	0.98
HLA-DMA*01:02	0.194	0.148	1.38 (0.72-2.67)	0.34		1.01 (0.49-2.09)	0.98
HLA-DMB							
HLA-DMB*01:01	0.565	0.493	1.33 (0.79-2.24)	0.28		1.38 (0.81-2.33)	0.23
HLA-DMB*01:02	0.129	0.191	0.63 (0.29-1.34)	0.23		0.61 (0.28-1.32)	0.21
HLA-DMB*01:03	0.290	0.307	0.93 (0.52-1.63)	0.79		0.90 (0.51-1.60)	0.72
HLA-DMB*01:07	0.016	0.008	1.99 (0.04-15.9)	0.43	*	—	—
HLA-DOA							
HLA-DOA*01:01	1.000	0.997	Inf	0.99		Inf	0.99
HLA-DOB							
HLA-DOB*01:01	0.790	0.768	1.14 (0.61-2.14)	0.69		1.16 (0.61-2.20)	0.65
HLA-DOB*01:02	0.177	0.182	0.97 (0.49-1.90)	0.93		1.01 (0.51-2.00)	0.97
HLA-DOB*01:04	0.032	0.050	0.63 (0.07-2.53)	0.76	*	—	—
HLA-DPA1							
HLA-DPA1*01:03	0.290	0.406	0.60 (0.34-1.05)	0.076		0.66 (0.37-1.16)	0.15
HLA-DPA1*02:01	0.161	0.171	0.93 (0.46-1.87)	0.84		1.00 (0.49-2.02)	1.0
HLA-DPA1*02:02	0.548	0.423	1.66 (0.99-2.78)	0.056		1.48 (0.87-2.51)	0.15
HLA-DPB1							
HLA-DPB1*02:01	0.161	0.220	0.68 (0.34-1.36)	0.28		0.76 (0.37-1.52)	0.43
HLA-DPB1*02:02	0.032	0.044	0.72 (0.08-2.90)	1.0	*	—	—
HLA-DPB1*03:01	0.016	0.055	0.28 (0.01-1.72)	0.25	*	—	—
HLA-DPB1*04:01	0.016	0.057	0.27 (0.01-1.64)	0.25	*	—	—
HLA-DPB1*04:02	0.113	0.093	1.24 (0.55-2.81)	0.61		1.37 (0.60-3.12)	0.46
HLA-DPB1*05:01	0.516	0.356	1.93 (1.15-3.24)	0.012		1.75 (1.03-2.97)	0.038
HLA-DPB1*09:01	0.097	0.118	0.80 (0.34-1.91)	0.62		0.77 (0.32-1.86)	0.56
HLA-DPB1*13:01	0.048	0.015	3.30 (0.59-12.5)	0.087	*	—	—
HLA-DQA1							
HLA-DQA1*01:01	0.129	0.058	2.39 (1.08-5.30)	0.031		2.65 (1.19-5.90)	0.017
HLA-DQA1*01:02	0.145	0.156	0.92 (0.44-1.90)	0.82		1.02 (0.49-2.12)	0.97
HLA-DQA1*01:03	0.177	0.204	0.84 (0.43-1.65)	0.62		0.94 (0.47-1.85)	0.85
HLA-DQA1*01:04	0.032	0.063	0.50 (0.06-1.97)	0.58	*	—	—
HLA-DQA1*03:01	0.065	0.089	0.71 (0.18-2.00)	0.65	*	—	—
HLA-DQA1*03:02	0.048	0.147	0.30 (0.06-0.93)	0.035	*	—	—
HLA-DQA1*03:03	0.177	0.141	1.31 (0.67-2.59)	0.43		1.46 (0.74-2.91)	0.28
HLA-DQA1*04:01	0.032	0.034	0.95 (0.11-3.92)	1.0	*	—	—
HLA-DQA1*05:03	0.097	0.015	6.96 (2.55-19.0)	$1.5{\times}10^{-4}$		—	—
HLA-DQA1*05:05	0.097	0.043	2.38 (0.96-5.87)	0.060		2.62 (1.06-6.50)	0.038
HLA-DQB1							
HLA-DQB1*03:01	0.210	0.104	2.29 (1.20-4.39)	0.012		2.57 (1.33-4.96)	0.0050
HLA-DQB1*03:02	0.097	0.098	0.99 (0.41-2.36)	0.98		0.89 (0.36-2.16)	0.79
HLA-DQB1*03:03	0.048	0.149	0.29 (0.06-0.91)	0.024	*	—	—
HLA-DQB1*04:01	0.145	0.100	1.52 (0.73-3.20)	0.26		1.41 (0.66-3.00)	0.38
HLA-DQB1*04:02	0.016	0.048	0.33 (0.01-2.00)	0.35	*	—	—
HLA-DQB1*05:01	0.129	0.063	2.21 (1.00-4.87)	0.050		2.15 (0.96-4.82)	0.062
HLA-DQB1*05:02	0.016	0.028	0.57 (0.01-3.62)	1.0	*	—	—
HLA-DQB1*05:03	0.032	0.044	0.72 (0.08-2.90)	1.0	*	—	—
HLA-DQB1*06:01	0.177	0.199	0.87 (0.44-1.70)	0.68		0.88 (0.45-1.74)	0.72
HLA-DQB1*06:02	0.097	0.068	1.48 (0.61-3.57)	0.39		1.52 (0.62-3.71)	0.36
HLA-DQB1*06:04	0.032	0.076	0.41 (0.05-1.60)	0.31	*	—	—
HLA-DRA							
HLA-DRA*01:01	0.597	0.579	1.08 (0.64-1.82)	0.79		1.33 (0.76-2.33)	0.32
HLA-DRA*01:02	0.403	0.421	0.93 (0.55-1.57)	0.79		0.75 (0.43-1.32)	0.32

HLA-DRB1							
HLA-DRB1*01:01	0.129	0.058	2.39 (1.08-5.30)	0.031		2.65 (1.19-5.90)	0.017
HLA-DRB1*04:01	0.016	0.013	1.26 (0.03-8.95)	0.57	*	—	—
HLA-DRB1*04:03	0.032	0.020	1.65 (0.18-7.21)	0.37	*	—	—
HLA-DRB1*04:05	0.145	0.120	1.24 (0.60-2.60)	0.56		1.27 (0.60-2.66)	0.53
HLA-DRB1*04:10	0.016	0.014	1.16 (0.03-8.05)	0.60	*	—	—
HLA-DRB1*08:02	0.048	0.056	0.86 (0.17-2.79)	1.0	*	—	—
HLA-DRB1*08:03	0.065	0.079	0.80 (0.21-2.27)	1.0	*	—	—
HLA-DRB1*09:01	0.016	0.135	0.10 (0.003-0.62)	0.0027	*	—	—
HLA-DRB1*10:01	0.016	0.003	4.66 (0.09-59.0)	0.24	*	—	—
HLA-DRB1*11:01	0.065	0.027	2.50 (0.61-7.67)	0.10	*	—	—
HLA-DRB1*12:01	0.016	0.030	0.52 (0.01-3.31)	1.0	*	—	—
HLA-DRB1*13:02	0.032	0.078	0.39 (0.05-1.55)	0.31	*	—	—
HLA-DRB1*14:05	0.032	0.027	1.21 (0.14-5.09)	0.68	*	—	—
HLA-DRB1*14:06	0.081	0.012	7.40(1.92-24.8)	0.0021	*	—	—
HLA-DRB1*14:54	0.016	0.036	0.44 (0.01-2.72)	0.72	*	—	—
HLA-DRB1*15:01	0.129	0.072	1.90 (0.87-4.18)	0.11		1.98 (0.90-4.39)	0.092
HLA-DRB1*15:02	0.129	0.120	1.09 (0.50-2.35)	0.83		1.10 (0.51-2.40)	0.81
HLA-DRB1*16:02	0.016	0.007	2.32 (0.05-19.6)	0.39	*	—	—
HLA-E							
HLA-E*01:01	0.290	0.307	0.93 (0.53-1.63)	0.79		1.02 (0.58-1.82)	0.93
HLA-E*01:03	0.710	0.694	1.08 (0.61-1.91)	0.79		0.98 (0.55-1.74)	0.93
HLA-F							
HLA-F*01:01	1.000	0.980	Inf	0.98		Inf	0.98
HLA-G							
HLA-G*01:01	0.597	0.511	1.42 (0.84-2.40)	0.19		1.45 (0.85-2.47)	0.17
HLA-G*01:03	0.016	0.001	13.9 (0.18-1093)	0.13	*	—	—
HLA-G*01:04	0.387	0.479	0.69 (0.41-1.17)	0.16		0.69 (0.41-1.18)	0.18

* Fisher's exact test was used for association analysis, otherwise logistic regression was used.

Table S3-1. Results of the HLA amino acid association analysis (multiple sclerosis)

Variant			Frequency					
	Position	amino acid	MS (n=45)	Control (n=429)	OR (95%CI)	*P*	Fisher	Omnibus *P*
**HLA-β**	-23	L	0.256	0.162	1.78 (1.07-2.95)	0.027		0.033
**(128amino acid)**	-23	R	0.744	0.838	0.56 (0.34-0.94)	0.027		
	-21	M	0.256	0.162	1.78 (1.07-2.95)	0.027		0.033
	-21	T	0.744	0.838	0.56 (0.34-0.94)	0.027		
	-16	L	0.233	0.353	0.56 (0.34-0.93)	0.024		0.019
	-16	V	0.767	0.647	1.79 (1.08-2.98)	0.024		
	-11	S	0.567	0.379	2.14 (1.38-3.33)	6.6×10^{-4}		6.2×10^{-4}
	-11	W	0.433	0.621	0.47 (0.30-0.72)	6.6×10^{-4}		
	-10	A	0.322	0.221	1.67 (1.04-2.68)	0.032		0.037
	-10	G	0.678	0.779	0.60 (0.37-0.96)	0.032		
	-8	L	0.656	0.499	1.91 (1.21-3.01)	0.0052		0.0043
	-8	V	0.344	0.501	0.52 (0.33-0.82)	0.0052		
	9	H	0.178	0.199	0.87 (0.49-1.53)	0.63		0.62
	9	Y	0.822	0.801	1.15 (0.65-2.03)	0.63		
	11	A	0.633	0.702	0.73 (0.47-1.16)	0.18		0.19
	11	S	0.367	0.298	1.36 (0.87-2.14)	0.18		
	12	M	0.633	0.702	0.73 (0.47-1.16)	0.18		0.19
	12	V	0.367	0.298	1.36 (0.87-2.14)	0.18		
	24	A	0.533	0.527	1.03 (0.66-1.59)	0.91		0.14
	24	S	0.256	0.184	1.52 (0.92-2.52)	0.10		
	24	T	0.211	0.289	0.66 (0.39-1.12)	0.12		
	32	L	0.211	0.276	0.70 (0.41-1.19)	0.19		0.18
	32	Q	0.789	0.724	1.43 (0.84-2.42)	0.19		
	41	A	0.789	0.712	1.51 (0.89-2.56)	0.13		0.11
	41	T	0.211	0.288	0.66 (0.39-1.12)	0.13		
	45	E	0.278	0.233	1.27 (0.78-2.06)	0.34		0.11
	45	G	0.067	0.069	0.97 (0.41-2.31)	0.94		
	45	K	0.211	0.275	0.71 (0.42-1.20)	0.19		
	45	M	0.244	0.152	1.81 (1.08-3.03)	0.024		
	45	T	0.200	0.272	0.67 (0.39-1.15)	0.15		
	46	A	0.244	0.152	1.81 (1.08-3.03)	0.024		0.030
	46	E	0.756	0.849	0.55 (0.33-0.92)	0.024		
	52	I	0.933	0.931	1.03 (0.43-2.47)	0.94		0.94
	52	V	0.067	0.069	0.97 (0.41-2.31)	0.94		
	62	G	0.022	0.005	4.84 (0.43-34.3)	0.10	*	0.11
	62	R	0.978	0.995	0.21 (0.04-1.14)	0.070		
	63	E	0.533	0.599	0.76 (0.49-1.18)	0.23		0.23
	63	N	0.467	0.401	1.31 (0.85-2.02)	0.23		
	65	Q	0.978	0.995	0.21 (0.04-1.14)	0.070		0.11
	65	R	0.022	0.005	4.84 (0.43-34.3)	0.10	*	
	66	I	0.956	0.949	1.16 (0.41-3.31)	0.78		0.14
	66	K	0.022	0.047	0.47 (0.05-1.85)	0.42	*	
	66	N	0.022	0.005	4.84 (0.43-34.3)	0.10	*	
	67	C	0.122	0.070	1.85 (0.94-3.67)	0.077		0.22
	67	F	0.144	0.153	0.94 (0.51-1.74)	0.84		
	67	M	0.022	0.005	4.84 (0.43-34.3)	0.10	*	
	67	S	0.489	0.548	0.79 (0.51-1.22)	0.29		
	67	Y	0.222	0.225	0.98 (0.58-1.66)	0.95		
	69	A	0.200	0.181	1.13 (0.66-1.96)	0.65		0.48
	69	R	0.022	0.047	0.47 (0.05-1.85)	0.42	*	
	69	T	0.778	0.773	1.03 (0.61-1.73)	0.91		
	70	N	0.778	0.773	1.03 (0.61-1.73)	0.91		0.23
	70	Q	0.200	0.221	0.88 (0.51-1.51)	0.64		
	70	S	0.022	0.005	4.84 (0.43-34.3)	0.10	*	
	71	A	0.222	0.227	0.97 (0.58-1.64)	0.91		0.91
	71	T	0.778	0.773	1.03 (0.61-1.73)	0.91		
	74	D	0.322	0.273	1.27 (0.79-2.02)	0.32		0.33
	74	Y	0.678	0.727	0.79 (0.49-1.26)	0.32		
	76	E	0.978	0.953	2.15 (0.51-9.05)	0.30		0.24
	76	V	0.022	0.047	0.47 (0.05-1.85)	0.42	*	
	77	N	0.200	0.322	0.53 (0.31-0.90)	0.019		0.0093
	77	S	0.800	0.675	1.93 (1.13-3.29)	0.016		
	80	I	0.167	0.225	0.69 (0.39-1.23)	0.21		0.015
	80	N	0.800	0.674	1.94 (1.13-3.31)	0.016		
	80	T	0.033	0.101	0.31 (0.06-0.96)	0.036	*	
	81	A	0.200	0.322	0.53 (0.31-0.90)	0.019		0.014
	81	L	0.800	0.678	1.90 (1.11-3.24)	0.019		
	82	L	0.200	0.326	0.52 (0.30-0.88)	0.016		0.011
	82	R	0.800	0.674	1.94 (1.13-3.31)	0.016		
	83	G	0.800	0.674	1.94 (1.13-3.31)	0.016		0.011
	83	R	0.200	0.326	0.52 (0.30-0.88)	0.016		
	94	I	0.100	0.157	0.60 (0.29-1.21)	0.15		0.13
	94	T	0.900	0.843	1.68 (0.82-3.43)	0.15		
	95	I	0.100	0.161	0.58 (0.28-1.18)	0.13		0.0066
	95	L	0.633	0.459	2.03 (1.30-3.19)	0.0019		
	95	W	0.267	0.380	0.59 (0.36-0.97)	0.036		
	97	R	0.567	0.439	1.67 (1.08-2.59)	0.022		0.047
	97	S	0.167	0.180	0.91 (0.51-1.63)	0.76		
	97	T	0.267	0.380	0.59 (0.36-0.97)	0.036		
	103	L	0.156	0.196	0.76 (0.42-1.37)	0.36		0.35
	103	V	0.844	0.804	1.32 (0.73-2.40)	0.36		
	113	H	0.967	0.914	2.74 (0.85-8.86)	0.093		0.052
	113	Y	0.033	0.086	0.37 (0.07-1.15)	0.10	*	
	114	D	0.422	0.365	1.27 (0.82-1.98)	0.28		0.25
	114	N	0.578	0.634	0.79 (0.51-1.23)	0.29		
	116	D	0.033	0.082	0.39 (0.08-1.22)	0.14	*	0.0043
	116	F	0.156	0.069	2.49 (1.33-4.68)	0.0044		
	116	L	0.089	0.132	0.64 (0.30-1.37)	0.25		
	116	S	0.322	0.225	1.64 (1.02-2.62)	0.040		
	116	Y	0.400	0.493	0.69 (0.44-1.07)	0.094		
	131	R	0.278	0.291	0.94 (0.58-1.52)	0.79		0.79
	131	S	0.722	0.709	1.07 (0.66-1.73)	0.79		
	143	S	0.100	0.097	1.04 (0.50-2.14)	0.92		0.92

	143	T	0.900	0.903	0.96 (0.47-1.99)	0.92		
	147	L	0.100	0.097	1.04 (0.50-2.14)	0.92		0.92
	147	W	0.900	0.903	0.96 (0.47-1.99)	0.92		
	152	E	0.444	0.422	1.10 (0.71-1.70)	0.68		0.68
	152	V	0.556	0.578	0.91 (0.59-1.41)	0.68		
	156	L	0.689	0.795	0.57 (0.35-0.92)	0.021		0.018
	156	R	0.067	0.059	1.13 (0.47-2.71)	0.78		
	156	W	0.244	0.138	2.03 (1.21-3.41)	0.0075		
	158	A	0.844	0.935	0.38 (0.20-0.71)	0.0026		0.0053
	158	T	0.156	0.065	2.64 (1.40-4.96)	0.0026		
	163	E	0.278	0.305	0.87 (0.54-1.42)	0.59		0.28
	163	L	0.478	0.521	0.84 (0.54-1.30)	0.44		
	163	T	0.244	0.174	1.54 (0.92-2.57)	0.099		
	167	S	0.033	0.080	0.39 (0.08-1.24)	0.14	*	0.076
	167	W	0.967	0.920	2.54 (0.78-8.23)	0.12		
	171	H	0.133	0.202	0.61 (0.32-1.14)	0.12		0.10
	171	Y	0.867	0.798	1.64 (0.87-3.08)	0.12		
	177	D	0.167	0.156	1.08 (0.60-1.94)	0.79		0.80
	177	E	0.833	0.844	0.93 (0.52-1.66)	0.79		
	178	K	0.167	0.156	1.08 (0.60-1.94)	0.79		0.80
	178	T	0.833	0.844	0.93 (0.52-1.66)	0.79		
	180	E	0.167	0.156	1.08 (0.60-1.94)	0.79		0.80
	180	Q	0.833	0.844	0.93 (0.52-1.66)	0.79		
	194	I	0.800	0.732	1.46 (0.86-2.51)	0.16		0.15
	194	V	0.200	0.268	0.68 (0.40-1.17)	0.16		
	199	A	0.967	0.920	2.54 (0.78-8.23)	0.12		0.076
	199	V	0.033	0.080	0.39 (0.08-1.24)	0.14	*	
	245	A	0.967	0.963	1.12 (0.34-3.74)	0.85		0.85
	245	T	0.033	0.037	0.89 (0.17-2.94)	1.0	*	
	282	I	0.533	0.548	0.94 (0.61-1.46)	0.79		0.60
	282	V	0.467	0.451	1.06 (0.69-1.65)	0.78		
	305	A	0.467	0.451	1.06 (0.69-1.65)	0.78		0.60
	305	T	0.533	0.548	0.94 (0.61-1.46)	0.79		
	325	C	0.311	0.386	0.72 (0.45-1.15)	0.17		0.14
	325	S	0.689	0.613	1.40 (0.88-2.23)	0.16		
**HLA-DRβ1**	-25	K	0.689	0.598	1.49 (0.93-2.37)	0.094		0.028
**(114 amino acid)**	-25	R	0.311	0.388	0.71 (0.45-1.14)	0.15		
	-24	F	0.311	0.203	1.78 (1.10-2.86)	0.018		0.0067
	-24	L	0.689	0.783	0.61 (0.38-0.99)	0.043		
	-17	A	0.711	0.728	0.92 (0.57-1.48)	0.73		0.099
	-17	T	0.289	0.258	1.17 (0.72-1.89)	0.52		
	-16	A	0.689	0.598	1.49 (0.93-2.37)	0.094		0.026
	-16	V	0.311	0.387	0.72 (0.45-1.14)	0.16		
	-1	A	0.756	0.794	0.80 (0.48-1.34)	0.40		0.058
	-1	S	0.244	0.192	1.36 (0.82-2.26)	0.24		
	4	Q	0.089	0.138	0.61 (0.29-1.30)	0.20		0.047
	4	R	0.911	0.851	1.80 (0.85-3.80)	0.13		
	9	E	0.622	0.596	1.12 (0.72-1.75)	0.62		0.17
	9	K	0.089	0.136	0.62 (0.29-1.31)	0.21		
	9	W	0.289	0.259	1.16 (0.72-1.88)	0.54		
	10	Q	0.689	0.598	1.49 (0.93-2.37)	0.094		0.031
	10	Y	0.311	0.389	0.71 (0.44-1.13)	0.15		
	11	D	0.089	0.136	0.62 (0.29-1.31)	0.21		0.050
	11	L	0.044	0.058	0.75 (0.19-2.12)	0.81	*	
	11	P	0.244	0.199	1.30 (0.78-2.16)	0.31		
	11	S	0.311	0.389	0.71 (0.44-1.13)	0.15		
	11	V	0.311	0.206	1.74 (1.08-2.80)	0.023		
	12	K	0.689	0.601	1.47 (0.92-2.34)	0.11		0.046
	12	T	0.311	0.389	0.71 (0.44-1.13)	0.15		
	13	F	0.133	0.198	0.62 (0.33-1.17)	0.14		0.038
	13	G	0.167	0.189	0.86 (0.48-1.53)	0.61		
	13	H	0.311	0.203	1.78 (1.10-2.86)	0.018		
	13	R	0.244	0.199	1.30 (0.78-2.16)	0.31		
	13	S	0.144	0.201	0.67 (0.37-1.24)	0.20		
	16	H	0.789	0.775	1.08 (0.64-1.84)	0.76		0.27
	16	Q	0.044	0.027	1.69 (0.41-5.10)	0.31	*	
	16	Y	0.167	0.189	0.86 (0.48-1.53)	0.61		
	26	F	0.833	0.738	1.78 (1.00-3.16)	0.05		0.070
	26	L	0.078	0.115	0.65 (0.29-1.44)	0.28		
	26	Y	0.089	0.138	0.61 (0.29-1.30)	0.20		
	28	D	0.811	0.712	1.74 (1.00-3.00)	0.049		0.068
	28	E	0.100	0.142	0.67 (0.33-1.37)	0.27		
	28	H	0.089	0.136	0.62 (0.29-1.31)	0.21		
	30	C	0.044	0.058	0.75 (0.19-2.12)	0.81	*	0.11
	30	G	0.089	0.136	0.62 (0.29-1.31)	0.21		
	30	H	0.033	0.054	0.61 (0.12-1.96)	0.62	*	
	30	Y	0.833	0.738	1.78 (1.00-3.16)	0.05		
	31	F	0.867	0.793	1.70 (0.91-3.19)	0.098		0.033
	31	I	0.133	0.195	0.64 (0.34-1.20)	0.16		
	32	H	0.189	0.228	0.79 (0.45-1.37)	0.39		0.12
	32	Y	0.811	0.762	1.34 (0.77-2.32)	0.30		
	33	H	0.311	0.203	1.78 (1.10-2.86)	0.018		0.010
	33	N	0.689	0.788	0.60 (0.37-0.96)	0.033		
	37	F	0.078	0.064	1.23 (0.54-2.79)	0.62		0.19
	37	L	0.033	0.054	0.61 (0.12-1.96)	0.62	*	
	37	N	0.167	0.245	0.62 (0.35-1.10)	0.10		
	37	S	0.356	0.291	1.34 (0.85-2.12)	0.21		
	37	Y	0.367	0.337	1.14 (0.73-1.79)	0.57		
	38	L	0.033	0.054	0.61 (0.12-1.96)	0.62	*	0.082
	38	V	0.967	0.934	2.06 (0.63-6.73)	0.23		
	47	F	0.333	0.356	0.91 (0.57-1.44)	0.68		0.18
	47	Y	0.667	0.635	1.15 (0.73-1.82)	0.55		
	57	A	0.022	0.036	0.61 (0.07-2.45)	0.76	*	0.057
	57	D	0.544	0.549	0.98 (0.63-1.52)	0.93		
	57	S	0.311	0.215	1.65 (1.03-2.66)	0.038		
	57	V	0.122	0.191	0.59 (0.31-1.13)	0.11		
	60	H	0.022	0.036	0.61 (0.07-2.45)	0.76	*	0.070

	60	S	0.122	0.191	0.59 (0.31-1.13)	0.11		
	60	Y	0.856	0.763	1.84 (1.00-3.37)	0.050		
	67	F	0.144	0.240	0.53 (0.29-0.98)	0.043		0.033
	67	I	0.411	0.389	1.10 (0.70-1.70)	0.69		
	67	L	0.444	0.361	1.41 (0.91-2.19)	0.12		
	70	D	0.244	0.322	0.68 (0.41-1.13)	0.14		0.029
	70	Q	0.600	0.466	1.72 (1.10-2.67)	0.017		
	70	R	0.156	0.203	0.72 (0.40-1.31)	0.29		
	71	A	0.244	0.192	1.36 (0.82-2.26)	0.24		0.055
	71	E	0.056	0.082	0.66 (0.20-1.69)	0.54	*	
	71	R	0.700	0.703	0.99 (0.61-1.59)	0.96		
	74	A	0.611	0.584	1.12 (0.72-1.75)	0.62		0.32
	74	E	0.233	0.255	0.89 (0.53-1.48)	0.65		
	74	L	0.156	0.149	1.05 (0.58-1.91)	0.87		
	78	V	0.089	0.138	0.61 (0.29-1.30)	0.20		0.060
	78	Y	0.911	0.853	1.76 (0.83-3.74)	0.14		
	85	A	0.033	0.054	0.61 (0.12-1.96)	0.62	*	0.12
	85	V	0.967	0.937	1.95 (0.60-6.36)	0.27		
	86	G	0.611	0.717	0.62 (0.40-0.97)	0.038		0.012
	86	V	0.389	0.274	1.69 (1.08-2.64)	0.023		
	96	E	0.044	0.058	0.75 (0.19-2.12)	0.81	*	0.023
	96	H	0.400	0.525	0.60 (0.39-0.94)	0.026		
	96	Q	0.244	0.203	1.27 (0.76-2.12)	0.35		
	96	Y	0.311	0.203	1.78 (1.10-2.86)	0.018		
	98	E	0.400	0.340	1.29 (0.83-2.02)	0.26		0.079
	98	K	0.600	0.648	0.81 (0.52-1.27)	0.37		
	104	A	0.400	0.340	1.29 (0.83-2.02)	0.26		0.079
	104	S	0.600	0.648	0.81 (0.52-1.27)	0.37		
	120	N	0.311	0.206	1.74 (1.08-2.80)	0.023		0.010
	120	S	0.689	0.782	0.62 (0.38-0.99)	0.046		
	133	L	0.244	0.199	1.30 (0.78-2.16)	0.31		0.089
	133	R	0.756	0.789	0.83 (0.50-1.37)	0.46		
	140	A	0.378	0.395	0.93 (0.59-1.45)	0.75		0.14
	140	T	0.622	0.593	1.13 (0.72-1.77)	0.59		
	142	M	0.244	0.199	1.30 (0.78-2.16)	0.31		0.089
	142	V	0.756	0.789	0.83 (0.50-1.37)	0.46		
	149	H	0.311	0.385	0.72 (0.45-1.15)	0.17		0.041
	149	Q	0.689	0.604	1.45 (0.91-2.32)	0.12		
	180	L	0.311	0.203	1.78 (1.10-2.86)	0.018		0.0082
	180	V	0.689	0.786	0.60 (0.38-0.97)	0.038		
	181	M	0.089	0.141	0.59 (0.28-1.26)	0.17		0.040
	181	T	0.911	0.847	1.85 (0.87-3.91)	0.11		
	189	R	0.867	0.853	1.12 (0.59-2.11)	0.73		0.16
	189	S	0.133	0.135	0.98 (0.52-1.86)	0.96		
	233	R	0.178	0.249	0.65 (0.37-1.14)	0.13		0.025
	233	T	0.822	0.737	1.65 (0.94-2.90)	0.079		
	235	F	0.978	0.973	1.21 (0.28-5.23)	0.80		0.092
	235	R	0.022	0.013	1.75 (0.19-8.20)	0.35	*	
	236	L	0.978	0.973	1.21 (0.28-5.23)	0.80		0.19
	236	deletion	0.022	0.017	1.28 (0.14-5.63)	0.67	*	
	237	S	0.978	0.973	1.21 (0.28-5.23)	0.80		0.19
	237	deletion	0.022	0.017	1.28 (0.14-5.63)	0.67	*	
**HLA-DQβ1**	-32	M	0.967	0.910	2.86 (0.88-9.25)	0.079		0.032
**(164 amino acid)**	-32	deletion	0.033	0.084	0.38 (0.07-1.19)	0.10	*	
	-31	S	0.967	0.910	2.86 (0.88-9.25)	0.079		0.032
	-31	deletion	0.033	0.084	0.38 (0.07-1.19)	0.10	*	
	-30	W	0.967	0.910	2.86 (0.88-9.25)	0.079		0.032
	-30	deletion	0.033	0.084	0.38 (0.07-1.19)	0.10	*	
	-29	K	0.967	0.910	2.86 (0.88-9.25)	0.079		0.032
	-29	deletion	0.033	0.084	0.38 (0.07-1.19)	0.10	*	
	-28	K	0.967	0.910	2.86 (0.88-9.25)	0.079		0.032
	-28	deletion	0.033	0.084	0.38 (0.07-1.19)	0.10	*	
	-27	A	0.833	0.775	1.45 (0.81-2.58)	0.21		0.098
	-27	S	0.133	0.135	0.98 (0.52-1.86)	0.96		
	-27	deletion	0.033	0.084	0.38 (0.07-1.19)	0.10	*	
	-26	L	0.967	0.910	2.86 (0.88-9.25)	0.079		0.032
	-26	deletion	0.033	0.084	0.38 (0.07-1.19)	0.10	*	
	-25	R	0.967	0.910	2.86 (0.88-9.25)	0.079		0.032
	-25	deletion	0.033	0.084	0.38 (0.07-1.19)	0.10	*	
	-24	I	0.967	0.910	2.86 (0.88-9.25)	0.079		0.032
	-24	deletion	0.033	0.084	0.38 (0.07-1.19)	0.10	*	
	-23	P	0.967	0.910	2.86 (0.88-9.25)	0.079		0.032
	-23	deletion	0.033	0.084	0.38 (0.07-1.19)	0.10	*	
	-22	G	0.967	0.910	2.86 (0.88-9.25)	0.079		0.032
	-22	deletion	0.033	0.084	0.38 (0.07-1.19)	0.10	*	
	-21	D	0.356	0.210	2.08 (1.31-3.30)	0.0019		0.0025
	-21	G	0.611	0.701	0.67 (0.43-1.05)	0.082		
	-21	deletion	0.033	0.084	0.38 (0.07-1.19)	0.10	*	
	-20	L	0.967	0.910	2.86 (0.88-9.25)	0.079		0.032
	-20	deletion	0.033	0.084	0.38 (0.07-1.19)	0.10	*	
	-19	R	0.967	0.910	2.86 (0.88-9.25)	0.079		0.032
	-19	deletion	0.033	0.084	0.38 (0.07-1.19)	0.10	*	
	-18	A	0.156	0.305	0.42 (0.23-0.75)	0.0038		2.2×10^{-4}
	-18	V	0.811	0.605	2.80 (1.63-4.84)	2.1×10^{-4}		
	-18	deletion	0.033	0.084	0.38 (0.07-1.19)	0.10	*	
	-17	A	0.844	0.711	2.21 (1.22-3.98)	0.0084		0.011
	-17	P	0.122	0.199	0.56 (0.29-1.07)	0.081		
	-17	deletion	0.033	0.084	0.38 (0.07-1.19)	0.10	*	
	-16	T	0.967	0.910	2.86 (0.88-9.25)	0.079		0.032
	-16	deletion	0.033	0.084	0.38 (0.07-1.19)	0.10	*	
	-15	V	0.967	0.910	2.86 (0.88-9.25)	0.079		0.032
	-15	deletion	0.033	0.084	0.38 (0.07-1.19)	0.10	*	
	-14	T	0.967	0.910	2.86 (0.88-9.25)	0.079		0.032
	-14	deletion	0.033	0.084	0.38 (0.07-1.19)	0.10	*	
	-13	L	0.967	0.910	2.86 (0.88-9.25)	0.079		0.032
	-13	deletion	0.033	0.084	0.38 (0.07-1.19)	0.10	*	
	-12	M	0.967	0.910	2.86 (0.88-9.25)	0.079		0.032

-12	deletion	0.033	0.084	0.38 (0.12-1.22)	0.10	*	
-11	L	0.967	0.910	2.86 (0.88-9.25)	0.079		0.032
-11	deletion	0.033	0.084	0.38 (0.07-1.19)	0.10	*	
-10	A	0.967	0.908	2.94 (0.91-9.51)	0.072		0.025
-10	deletion	0.033	0.084	0.38 (0.07-1.19)	0.10	*	
-9	I	0.133	0.135	0.98 (0.52-1.86)	0.96		0.098
-9	M	0.833	0.775	1.45 (0.81-2.58)	0.21		
-9	deletion	0.033	0.084	0.38 (0.07-1.19)	0.10	*	
-8	L	0.967	0.910	2.86 (0.88-9.25)	0.079		0.032
-8	deletion	0.033	0.084	0.38 (0.07-1.19)	0.10	*	
-7	S	0.967	0.910	2.86 (0.88-9.25)	0.079		0.032
-7	deletion	0.033	0.084	0.38 (0.07-1.19)	0.10	*	
-6	S	0.356	0.210	2.08 (1.31-3.30)	0.0019		0.0025
-6	T	0.611	0.701	0.67 (0.43-1.05)	0.082		
-6	deletion	0.033	0.084	0.38 (0.07-1.19)	0.10	*	
-5	L	0.222	0.075	3.54 (2.03-6.20)	8.9×10^{-6}		1.7×10^{-4}
-5	P	0.611	0.701	0.67 (0.43-1.05)	0.082		
-5	S	0.133	0.135	0.98 (0.52-1.86)	0.96		
-5	deletion	0.033	0.084	0.38 (0.07-1.19)	0.10	*	
-4	L	0.356	0.210	2.08 (1.31-3.30)	0.0019		0.0025
-4	V	0.611	0.701	0.67 (0.43-1.05)	0.082		
-4	deletion	0.033	0.084	0.38 (0.07-1.19)	0.10	*	
-3	A	0.967	0.910	2.86 (0.88-9.25)	0.079		0.032
-3	deletion	0.033	0.084	0.38 (0.07-1.19)	0.10	*	
-2	E	0.967	0.910	2.86 (0.88-9.25)	0.079		0.032
-2	deletion	0.033	0.084	0.38 (0.07-1.19)	0.10	*	
-1	G	0.967	0.910	2.86 (0.88-9.25)	0.079		0.032
-1	deletion	0.033	0.084	0.38 (0.07-1.19)	0.10	*	
3	P	0.122	0.199	0.56 (0.29-1.07)	0.081		0.033
3	S	0.878	0.795	1.85 (0.97-3.56)	0.064		
9	F	0.489	0.216	3.48 (2.23-5.43)	3.7×10^{-8}		3.6×10^{-7}
9	L	0.122	0.199	0.56 (0.29-1.07)	0.081		
9	Y	0.389	0.579	0.46 (0.30-0.72)	6.8×10^{-4}		
13	A	0.156	0.311	0.41 (0.23-0.73)	0.0028		6.0×10^{-4}
13	G	0.844	0.683	2.52 (1.40-4.54)	0.0021		
14	L	0.133	0.135	0.98 (0.52-1.86)	0.96		0.32
14	M	0.867	0.859	1.07 (0.56-2.02)	0.84		
23	L	0.222	0.100	2.56 (1.49-4.42)	7.0×10^{-4}		9.3×10^{-4}
23	R	0.778	0.894	0.42 (0.24-0.71)	0.0015		
26	G	0.422	0.283	1.85 (1.19-2.88)	0.0066		0.0010
26	L	0.422	0.400	1.10 (0.71-1.70)	0.68		
26	Y	0.156	0.311	0.41 (0.23-0.73)	0.0028		
30	H	0.167	0.215	0.73 (0.41-1.31)	0.29		0.10
30	Y	0.833	0.777	1.43 (0.80-2.55)	0.22		
37	D	0.122	0.199	0.56 (0.29-1.07)	0.081		0.026
37	Y	0.878	0.793	1.88 (0.98-3.61)	0.058		
38	A	0.744	0.657	1.52 (0.93-2.49)	0.098		0.056
38	V	0.256	0.337	0.68 (0.41-1.11)	0.12		
45	E	0.033	0.112	0.27 (0.05-0.85)	0.017	*	0.0046
45	G	0.967	0.882	3.87 (1.20-12.5)	0.023		
53	L	0.489	0.509	0.92 (0.60-1.42)	0.71		0.28
53	Q	0.511	0.485	1.11 (0.72-1.72)	0.64		
55	P	0.200	0.359	0.45 (0.26-0.76)	0.0031		6.5×10^{-4}
55	R	0.800	0.633	2.32 (1.36-3.96)	0.0020		
56	L	0.289	0.148	2.34 (1.43-3.83)	7.4×10^{-4}		8.3×10^{-4}
56	P	0.711	0.846	0.45 (0.27-0.73)	0.0014		
57	A	0.078	0.108	0.69 (0.31-1.55)	0.37		0.38
57	D	0.789	0.716	1.48 (0.88-2.52)	0.14		
57	S	0.022	0.028	0.79 (0.09-3.27)	1.0	*	
57	V	0.111	0.142	0.75 (0.38-1.50)	0.42		
66	D	0.411	0.350	1.30 (0.83-2.02)	0.25		0.14
66	E	0.589	0.645	0.79 (0.51-1.23)	0.30		
67	I	0.411	0.350	1.30 (0.83-2.02)	0.25		0.14
67	V	0.589	0.645	0.79 (0.51-1.23)	0.30		
70	E	0.289	0.148	2.34 (1.43-3.83)	7.4×10^{-4}		3.8×10^{-6}
70	G	0.333	0.206	1.92 (1.20-3.07)	0.0062		
70	R	0.378	0.640	0.34 (0.22-0.54)	2.7×10^{-6}		
71	A	0.133	0.135	0.98 (0.52-1.86)	0.96		0.0027
71	D	0.289	0.148	2.34 (1.43-3.83)	7.4×10^{-4}		
71	T	0.578	0.709	0.56 (0.36-0.88)	0.011		
74	E	0.578	0.709	0.56 (0.36-0.88)	0.011		0.0040
74	S	0.422	0.283	1.85 (1.19-2.88)	0.0066		
75	L	0.578	0.709	0.56 (0.36-0.88)	0.011		0.0056
75	V	0.422	0.286	1.83 (1.17-2.85)	0.0077		
77	R	0.133	0.138	0.96 (0.51-1.83)	0.91		0.31
77	T	0.867	0.857	1.09 (0.58-2.06)	0.80		
84	E	0.511	0.485	1.11 (0.72-1.72)	0.64		0.28
84	Q	0.489	0.509	0.92 (0.60-1.42)	0.71		
85	L	0.489	0.509	0.92 (0.60-1.42)	0.71		0.28
85	V	0.511	0.485	1.11 (0.72-1.72)	0.64		
86	A	0.456	0.406	1.23 (0.79-1.90)	0.36		0.33
86	E	0.489	0.509	0.92 (0.60-1.42)	0.71		
86	G	0.056	0.079	0.68 (0.21-1.74)	0.54	*	
87	F	0.322	0.270	1.28 (0.80-2.05)	0.30		0.35
87	L	0.489	0.509	0.92 (0.60-1.42)	0.71		
87	Y	0.189	0.215	0.85 (0.49-1.48)	0.57		
89	G	0.511	0.485	1.11 (0.72-1.72)	0.64		0.28
89	T	0.489	0.509	0.92 (0.60-1.42)	0.71		
90	I	0.511	0.485	1.11 (0.72-1.72)	0.64		0.28
90	T	0.489	0.509	0.92 (0.60-1.42)	0.71		
116	I	0.133	0.135	0.98 (0.52-1.86)	0.96		0.32
116	V	0.867	0.859	1.07 (0.56-2.02)	0.84		
125	A	0.489	0.509	0.92 (0.60-1.42)	0.71		0.54
125	G	0.378	0.350	1.13 (0.72-1.77)	0.60		
125	S	0.133	0.135	0.98 (0.52-1.86)	0.96		
126	H	0.022	0.028	0.79 (0.09-3.27)	1.0	*	0.29
126	Q	0.978	0.966	1.54 (0.36-6.56)	0.56		

130	Q	0.056	0.079	0.68 (0.21-1.74)	0.54	*	0.19
130	R	0.944	0.915	1.58 (0.62-4.02)	0.34		
140	A	0.511	0.487	1.10 (0.71-1.70)	0.67		0.28
140	T	0.489	0.507	0.93 (0.60-1.44)	0.74		
167	H	0.156	0.311	0.41 (0.23-0.73)	0.0028		6.0×10^{-4}
167	R	0.844	0.683	2.52 (1.40-4.54)	0.0021		
182	N	0.489	0.507	0.93 (0.60-1.44)	0.74		0.28
182	S	0.511	0.487	1.10 (0.71-1.70)	0.67		
185	I	0.456	0.395	1.28 (0.83-1.98)	0.27		0.14
185	T	0.544	0.599	0.80 (0.52-1.24)	0.32		
197	N	0.122	0.199	0.56 (0.29-1.07)	0.081		0.033
197	S	0.878	0.795	1.85 (0.97-3.56)	0.064		
203	I	0.611	0.701	0.67 (0.43-1.05)	0.082		0.014
203	V	0.389	0.286	1.59 (1.02-2.49)	0.042		
220	H	0.489	0.501	0.95 (0.62-1.47)	0.82		0.11
220	R	0.511	0.485	1.11 (0.72-1.72)	0.64		
221	H	0.489	0.501	0.95 (0.62-1.47)	0.82		0.11
221	Q	0.511	0.485	1.11 (0.72-1.72)	0.64		
224	Q	0.867	0.851	1.14 (0.60-2.15)	0.69		0.12
224	R	0.133	0.135	0.98 (0.52-1.86)	0.96		
230	A	0.822	0.751	1.54 (0.88-2.70)	0.13		0.076
230	P	0.178	0.244	0.67 (0.38-1.18)	0.17		

* Fisher's exact test was used for association analysis, otherwise logistic regression was used.

Table S3-2. Results of the conditional HLA DQβ1 association analysis (multiple sclerosis)

Variant			Frequency		Nominal analysis			Conditional association			
								HLA-DQβ1 (Phe9)		HLA-DRB1*15:01	
	Position	amino acid	NMO (n=31)	Control (n=429)	OR (95%CI)	*P*	Fisher	OR (95%CI)	*P*	OR (95%CI)	*P*
HLA-DQβ1	-32	M	0.967	0.910	2.86 (0.88-9.25)	0.079		1.85 (0.56-6.11)	0.31	2.63 (0.81-8.53)	0.11
(164 amino acid)	-32	deletion	0.033	0.084	0.38 (0.07-1.19)	0.10	*				
	-31	S	0.967	0.910	2.86 (0.88-9.25)	0.079		1.85 (0.56-6.11)	0.31	2.63 (0.81-8.53)	0.11
	-31	deletion	0.033	0.084	0.38 (0.07-1.19)	0.10	*				
	-30	W	0.967	0.910	2.86 (0.88-9.25)	0.079		1.85 (0.56-6.11)	0.31	2.63 (0.81-8.53)	0.11
	-30	deletion	0.033	0.084	0.38 (0.07-1.19)	0.10	*				
	-29	K	0.967	0.910	2.86 (0.88-9.25)	0.079		1.85 (0.56-6.11)	0.31	2.63 (0.81-8.53)	0.11
	-29	deletion	0.033	0.084	0.38 (0.07-1.19)	0.10	*				
	-28	K	0.967	0.910	2.86 (0.88-9.25)	0.079		1.85 (0.56-6.11)	0.31	2.63 (0.81-8.53)	0.11
	-28	deletion	0.033	0.084	0.38 (0.07-1.19)	0.10	*				
	-27	A	0.833	0.775	1.45 (0.81-2.58)	0.21		0.83 (0.44-1.57)	0.57	1.31 (0.73-2.35)	0.37
	-27	S	0.133	0.135	0.98 (0.52-1.86)	0.96		1.69 (0.85-3.37)	0.13	1.09 (0.57-2.07)	0.80
	-27	deletion	0.033	0.084	0.38 (0.07-1.19)	0.10	*				
	-26	L	0.967	0.910	2.86 (0.88-9.25)	0.079		1.85 (0.56-6.11)	0.31	2.63 (0.81-8.53)	0.11
	-26	deletion	0.033	0.084	0.38 (0.07-1.19)	0.10	*				
	-25	R	0.967	0.910	2.86 (0.88-9.25)	0.079		1.85 (0.56-6.11)	0.31	2.63 (0.81-8.53)	0.11
	-25	deletion	0.033	0.084	0.38 (0.07-1.19)	0.10	*				
	-24	I	0.967	0.910	2.86 (0.88-9.25)	0.079		1.85 (0.56-6.11)	0.31	2.63 (0.81-8.53)	0.11
	-24	deletion	0.033	0.084	0.38 (0.07-1.19)	0.10	*				
	-23	P	0.967	0.910	2.86 (0.88-9.25)	0.079		1.85 (0.56-6.11)	0.31	2.63 (0.81-8.53)	0.11
	-23	deletion	0.033	0.084	0.38 (0.07-1.19)	0.10	*				
	-22	G	0.967	0.910	2.86 (0.88-9.25)	0.079		1.85 (0.56-6.11)	0.31	2.63 (0.81-8.53)	0.11
	-22	deletion	0.033	0.084	0.38 (0.07-1.19)	0.10	*				
	-21	D	0.356	0.210	2.08 (1.31-3.30)	0.0019		1.73 (1.08-2.79)	0.023	1.52 (0.90-2.57)	0.12
	-21	G	0.611	0.701	0.67 (0.43-1.05)	0.082		0.69 (0.44-1.09)	0.11	0.91 (0.55-1.49)	0.71
	-21	deletion	0.033	0.084	0.38 (0.07-1.19)	0.10	*				
	-20	L	0.967	0.910	2.86 (0.88-9.25)	0.079		1.85 (0.56-6.11)	0.31	2.63 (0.81-8.53)	0.11
	-20	deletion	0.033	0.084	0.38 (0.07-1.19)	0.10	*				
	-19	R	0.967	0.910	2.86 (0.88-9.25)	0.079		1.85 (0.56-6.11)	0.31	2.63 (0.81-8.53)	0.11
	-19	deletion	0.033	0.084	0.38 (0.07-1.19)	0.10	*				
	-18	A	0.156	0.305	0.42 (0.23-0.75)	0.0038		0.69 (0.36-1.31)	0.25	0.47 (0.26-0.86)	0.014
	-18	V	0.811	0.605	2.80 (1.63-4.84)	0.00021		1.73 (0.93-3.21)	0.081	2.49 (1.43-4.33)	0.0012
	-18	deletion	0.033	0.084	0.38 (0.07-1.19)	0.10	*				
	-17	A	0.844	0.711	2.21 (1.22-3.98)	0.0084		1.33 (0.70-2.55)	0.38	2.00 (1.10-3.62)	0.023
	-17	P	0.122	0.199	0.56 (0.29-1.07)	0.081		0.92 (0.46-1.86)	0.82	0.62 (0.32-1.19)	0.15
	-17	deletion	0.033	0.084	0.38 (0.07-1.19)	0.10	*				
	-16	T	0.967	0.910	2.86 (0.88-9.25)	0.079		1.85 (0.56-6.11)	0.31	2.63 (0.81-8.53)	0.11
	-16	deletion	0.033	0.084	0.38 (0.07-1.19)	0.10	*				
	-15	V	0.967	0.910	2.86 (0.88-9.25)	0.079		1.85 (0.56-6.11)	0.31	2.63 (0.81-8.53)	0.11
	-15	deletion	0.033	0.084	0.38 (0.07-1.19)	0.10	*				
	-14	T	0.967	0.910	2.86 (0.88-9.25)	0.079		1.85 (0.56-6.11)	0.31	2.63 (0.81-8.53)	0.11
	-14	deletion	0.033	0.084	0.38 (0.07-1.19)	0.10	*				
	-13	L	0.967	0.910	2.86 (0.88-9.25)	0.079		1.85 (0.56-6.11)	0.31	2.63 (0.81-8.53)	0.11
	-13	deletion	0.033	0.084	0.38 (0.07-1.19)	0.10	*				
	-12	M	0.967	0.910	2.86 (0.88-9.25)	0.079		1.85 (0.56-6.11)	0.31	2.63 (0.81-8.53)	0.11
	-12	deletion	0.033	0.084	0.38 (0.12-1.22)	0.10	*				
	-11	L	0.967	0.910	2.86 (0.88-9.25)	0.079		1.85 (0.56-6.11)	0.31	2.63 (0.81-8.53)	0.11
	-11	deletion	0.033	0.084	0.38 (0.07-1.19)	0.10	*				
	-10	A	0.967	0.908	2.94 (0.91-9.51)	0.072		1.91 (0.58-6.29)	0.29	2.70 (0.83-8.76)	0.099
	-10	deletion	0.033	0.084	0.38 (0.07-1.19)	0.10	*				
	-9	I	0.133	0.135	0.98 (0.52-1.86)	0.96		1.69 (0.85-3.37)	0.13	1.09 (0.57-2.07)	0.80
	-9	M	0.833	0.775	1.45 (0.81-2.58)	0.21		0.83 (0.44-1.57)	0.57	1.31 (0.73-2.35)	0.37
	-9	deletion	0.033	0.084	0.38 (0.07-1.19)	0.10	*				
	-8	L	0.967	0.910	2.86 (0.88-9.25)	0.079		1.85 (0.56-6.11)	0.31	2.63 (0.81-8.53)	0.11
	-8	deletion	0.033	0.084	0.38 (0.07-1.19)	0.10	*				
	-7	S	0.967	0.910	2.86 (0.88-9.25)	0.079		1.85 (0.56-6.11)	0.31	2.63 (0.81-8.53)	0.11
	-7	deletion	0.033	0.084	0.38 (0.07-1.19)	0.10	*				
	-6	S	0.356	0.210	2.08 (1.31-3.30)	0.0019		1.73 (1.08-2.79)	0.023	1.52 (0.90-2.57)	0.12
	-6	T	0.611	0.701	0.67 (0.43-1.05)	0.082		0.69 (0.44-1.09)	0.11	0.91 (0.55-1.49)	0.71
	-6	deletion	0.033	0.084	0.38 (0.07-1.19)	0.10	*				
	-5	L	0.222	0.075	3.54 (2.03-6.20)	8.9×10^{-6}		1.74 (0.91-3.31)	0.093	2.28 (1.04-5.01)	0.040
	-5	P	0.611	0.701	0.67 (0.43-1.05)	0.082		0.69 (0.44-1.09)	0.11	0.91 (0.55-1.49)	0.71
	-5	S	0.133	0.135	0.98 (0.52-1.86)	0.96		1.69 (0.85-3.37)	0.13	1.09 (0.57-2.07)	0.80
	-5	deletion	0.033	0.084	0.38 (0.07-1.19)	0.10	*				
	-4	L	0.356	0.210	2.08 (1.31-3.30)	0.0019		1.73 (1.08-2.79)	0.023	1.52 (0.90-2.57)	0.12
	-4	V	0.611	0.701	0.67 (0.43-1.05)	0.082		0.69 (0.44-1.09)	0.11	0.91 (0.55-1.49)	0.71
	-4	deletion	0.033	0.084	0.38 (0.07-1.19)	0.10	*				
	-3	A	0.967	0.910	2.86 (0.88-9.25)	0.079		1.85 (0.56-6.11)	0.31	2.63 (0.81-8.53)	0.11
	-3	deletion	0.033	0.084	0.38 (0.07-1.19)	0.10	*				
	-2	E	0.967	0.910	2.86 (0.88-9.25)	0.079		1.85 (0.56-6.11)	0.31	2.63 (0.81-8.53)	0.11
	-2	deletion	0.033	0.084	0.38 (0.07-1.19)	0.10	*				
	-1	G	0.967	0.910	2.86 (0.88-9.25)	0.079		1.85 (0.56-6.11)	0.31	2.63 (0.81-8.53)	0.11
	-1	deletion	0.033	0.084	0.38 (0.07-1.19)	0.10	*				
	3	P	0.122	0.199	0.56 (0.29-1.07)	0.081		0.92 (0.46-1.86)	0.82	0.62 (0.32-1.19)	0.15
	3	S	0.878	0.795	1.85 (0.97-3.56)	0.064		1.13 (0.56-2.27)	0.74	1.68 (0.87-3.24)	0.12
	9	F	0.489	0.216	3.48 (2.23-5.43)	3.7×10^{-8}				2.91 (1.79-4.72)	1.5×10^{-5}
	9	L	0.122	0.199	0.56 (0.29-1.07)	0.081		0.92 (0.46-1.86)	0.82	0.62 (0.32-1.19)	0.15
	9	Y	0.389	0.579	0.46 (0.30-0.72)	0.00068		1.13 (0.56-2.27)	0.74	0.55 (0.35-0.88)	0.013
	13	A	0.156	0.311	0.41 (0.23-0.73)	0.0028		0.67 (0.35-1.27)	0.22	0.46 (0.25-0.83)	0.011
	13	G	0.844	0.683	2.52 (1.40-4.54)	0.0021		1.55 (0.81-2.96)	0.18	2.23 (1.23-4.05)	0.0082
	14	L	0.133	0.135	0.98 (0.52-1.86)	0.96		1.69 (0.85-3.37)	0.13	1.09 (0.57-2.07)	0.80
	14	M	0.867	0.859	1.07 (0.56-2.02)	0.84		0.62 (0.31-1.23)	0.17	0.96 (0.51-1.83)	0.91
	23	L	0.222	0.100	2.56 (1.49-4.42)	0.0007		0.96 (0.50-1.86)	0.90	2.80 (1.61-4.87)	2.8×10^{-4}
	23	R	0.778	0.894	0.42 (0.24-0.71)	0.0015		1.08 (0.56-2.07)	0.82	0.38 (0.22-0.66)	5.9×10^{-4}
	26	G	0.422	0.283	1.85 (1.19-2.88)	0.0066		1.03 (0.61-1.73)	0.91	2.14 (1.35-3.39)	0.0011
	26	L	0.422	0.400	1.10 (0.71-1.70)	0.68		1.23 (0.78-1.93)	0.37	0.87 (0.54-1.39)	0.55
	26	Y	0.156	0.311	0.41 (0.23-0.73)	0.0028		0.67 (0.35-1.27)	0.22	0.46 (0.25-0.83)	0.011
	30	H	0.167	0.215	0.73 (0.41-1.31)	0.29		1.29 (0.68-2.44)	0.44	0.80 (0.45-1.43)	0.45
	30	Y	0.833	0.777	1.43 (0.80-2.55)	0.22		0.82 (0.43-1.55)	0.54	1.31 (0.73-2.34)	0.37
	37	D	0.122	0.199	0.56 (0.29-1.07)	0.081		0.92 (0.46-1.86)	0.82	0.62 (0.32-1.19)	0.15
	37	Y	0.878	0.793	1.88 (0.98-3.61)	0.058		1.14 (0.57-2.30)	0.71	1.70 (0.88-3.29)	0.11
	38	A	0.744	0.657	1.52 (0.93-2.49)	0.098		0.78 (0.43-1.41)	0.40	1.35 (0.82-2.23)	0.24

38	V	0.256	0.337	0.68 (0.41-1.11)	0.12		1.33 (0.73-2.42)	0.35	0.76 (0.46-1.26)	0.28
45	E	0.033	0.112	0.27 (0.05-0.85)	0.017	*				
45	G	0.967	0.882	3.87 (1.20-12.5)	0.023		2.53 (0.77-8.31)	0.13	3.36 (1.04-10.9)	0.043
53	L	0.489	0.509	0.92 (0.60-1.42)	0.71		0.71 (0.45-1.12)	0.14	1.18 (0.74-1.88)	0.49
53	Q	0.511	0.485	1.11 (0.72-1.72)	0.64		1.43 (0.91-2.26)	0.12	0.87 (0.54-1.38)	0.55
55	P	0.200	0.359	0.45 (0.26-0.76)	0.0031		0.76 (0.41-1.40)	0.38	0.53 (0.30-0.91)	0.021
55	R	0.800	0.633	2.32 (1.36-3.96)	0.0020		1.37 (0.74-2.52)	0.31	1.97 (1.14-3.42)	0.015
56	L	0.289	0.148	2.34 (1.43-3.83)	0.00074		0.66 (0.34-1.30)	0.23	2.63 (1.59-4.36)	1.8×10^{-4}
56	P	0.711	0.846	0.45 (0.27-0.73)	0.0014		1.55 (0.80-3.03)	0.20	0.40 (0.24-0.66)	3.2×10^{-4}
57	A	0.078	0.108	0.69 (0.31-1.55)	0.37		1.12 (0.49-2.58)	0.79	0.77 (0.35-1.74)	0.53
57	D	0.789	0.716	1.48 (0.88-2.52)	0.14		0.81 (0.44-1.48)	0.49	1.33 (0.78-2.27)	0.30
57	S	0.022	0.028	0.79 (0.09-3.27)	1.0	*				
57	V	0.111	0.142	0.75 (0.38-1.50)	0.42		1.25 (0.61-2.60)	0.54	0.85 (0.42-1.69)	0.63
66	D	0.411	0.350	1.30 (0.83-2.02)	0.25		0.77 (0.47-1.26)	0.30	1.50 (0.95-2.37)	0.082
66	E	0.589	0.645	0.79 (0.51-1.23)	0.30		1.33 (0.81-2.18)	0.27	0.68 (0.43-1.08)	0.10
67	I	0.411	0.350	1.30 (0.83-2.02)	0.25		0.77 (0.47-1.26)	0.30	1.50 (0.95-2.37)	0.082
67	V	0.589	0.645	0.79 (0.51-1.23)	0.30		1.33 (0.81-2.18)	0.27	0.68 (0.43-1.08)	0.10
70	E	0.289	0.148	2.34 (1.43-3.83)	0.00074		0.66 (0.34-1.30)	0.23	2.63 (1.59-4.36)	1.8×10^{-4}
70	G	0.333	0.206	1.92 (1.20-3.07)	0.0062		1.58 (0.97-2.56)	0.065	1.36 (0.79-2.33)	0.26
70	R	0.378	0.640	0.34 (0.22-0.54)	0.0000027		0.64 (0.32-1.27)	0.20	0.40 (0.25-0.64)	1.5×10^{-4}
71	A	0.133	0.135	0.98 (0.52-1.86)	0.96		1.69 (0.85-3.37)	0.13	1.09 (0.57-2.07)	0.80
71	D	0.289	0.148	2.34 (1.43-3.83)	0.00074		0.66 (0.34-1.30)	0.23	2.63 (1.59-4.36)	1.8×10^{-4}
71	T	0.578	0.709	0.56 (0.36-0.88)	0.011		1.00 (0.60-1.68)	0.99	0.48 (0.31-0.77)	0.0020
74	E	0.578	0.709	0.56 (0.36-0.88)	0.011		1.00 (0.60-1.68)	0.99	0.48 (0.31-0.77)	0.0020
74	S	0.422	0.283	1.85 (1.19-2.88)	0.0066		1.03 (0.61-1.73)	0.91	2.14 (1.35-3.39)	0.0011
75	L	0.578	0.709	0.56 (0.36-0.88)	0.011		1.00 (0.60-1.68)	0.99	0.48 (0.31-0.77)	0.0020
75	V	0.422	0.286	1.83 (1.17-2.85)	0.0077		1.02 (0.61-1.71)	0.94	2.12 (1.34-3.35)	0.0013
77	R	0.133	0.138	0.96 (0.51-1.83)	0.91		1.66 (0.83-3.30)	0.15	1.07 (0.56-2.04)	0.84
77	T	0.867	0.857	1.09 (0.58-2.06)	0.80		0.63 (0.32-1.26)	0.19	0.98 (0.51-1.87)	0.95
84	E	0.511	0.485	1.11 (0.72-1.72)	0.64		1.43 (0.91-2.26)	0.12	0.87 (0.54-1.38)	0.55
84	Q	0.489	0.509	0.92 (0.60-1.42)	0.71		0.71 (0.45-1.12)	0.14	1.18 (0.74-1.88)	0.49
85	L	0.489	0.509	0.92 (0.60-1.42)	0.71		0.71 (0.45-1.12)	0.14	1.18 (0.74-1.88)	0.49
85	V	0.511	0.485	1.11 (0.72-1.72)	0.64		1.43 (0.91-2.26)	0.12	0.87 (0.54-1.38)	0.55
86	A	0.456	0.406	1.23 (0.79-1.90)	0.36		1.40 (0.90-2.20)	0.14	0.94 (0.58-1.51)	0.79
86	E	0.489	0.509	0.92 (0.60-1.42)	0.71		0.71 (0.45-1.12)	0.14	1.18 (0.74-1.88)	0.49
86	G	0.056	0.079	0.68 (0.21-1.74)	0.54	*				
87	F	0.322	0.270	1.28 (0.80-2.05)	0.30		1.17 (0.72-1.88)	0.52	0.88 (0.51-1.49)	0.62
87	L	0.489	0.509	0.92 (0.60-1.42)	0.71		0.71 (0.45-1.12)	0.14	1.18 (0.74-1.88)	0.49
87	Y	0.189	0.215	0.85 (0.49-1.48)	0.57		1.56 (0.84-2.90)	0.16	0.95 (0.54-1.66)	0.85
89	G	0.511	0.485	1.11 (0.72-1.72)	0.64		1.43 (0.91-2.26)	0.12	0.87 (0.54-1.38)	0.55
89	T	0.489	0.509	0.92 (0.60-1.42)	0.71		0.71 (0.45-1.12)	0.14	1.18 (0.74-1.88)	0.49
90	I	0.511	0.485	1.11 (0.72-1.72)	0.64		1.43 (0.91-2.26)	0.12	0.87 (0.54-1.38)	0.55
90	T	0.489	0.509	0.92 (0.60-1.42)	0.71		0.71 (0.45-1.12)	0.14	1.18 (0.74-1.88)	0.49
116	I	0.133	0.135	0.98 (0.52-1.86)	0.96		1.69 (0.85-3.37)	0.13	1.09 (0.57-2.07)	0.80
116	V	0.867	0.859	1.07 (0.56-2.02)	0.84		0.62 (0.31-1.23)	0.17	0.96 (0.51-1.83)	0.91
125	A	0.489	0.509	0.92 (0.60-1.42)	0.71		0.71 (0.45-1.12)	0.14	1.18 (0.74-1.88)	0.49
125	G	0.378	0.350	1.13 (0.72-1.77)	0.60		1.17 (0.74-1.85)	0.49	0.81 (0.49-1.34)	0.42
125	S	0.133	0.135	0.98 (0.52-1.86)	0.96		1.69 (0.85-3.37)	0.13	1.09 (0.57-2.07)	0.80
126	H	0.022	0.028	0.79 (0.09-3.27)	1.0	*				
126	Q	0.978	0.966	1.54 (0.36-6.56)	0.56		0.99 (0.23-4.29)	0.99	1.59 (0.37-6.85)	0.54
130	Q	0.056	0.079	0.68 (0.21-1.74)	0.54	*				
130	R	0.944	0.915	1.58 (0.62-4.02)	0.34		1.00 (0.38-2.60)	1.0	1.45 (0.57-3.70)	0.44
140	A	0.511	0.487	1.10 (0.71-1.70)	0.67		1.42 (0.90-2.25)	0.13	0.86 (0.54-1.37)	0.53
140	T	0.489	0.507	0.93 (0.60-1.44)	0.74		0.71 (0.45-1.13)	0.15	1.19 (0.75-1.89)	0.46
167	H	0.156	0.311	0.41 (0.23-0.73)	0.0028		0.67 (0.35-1.27)	0.22	0.46 (0.25-0.83)	0.011
167	R	0.844	0.683	2.52 (1.40-4.54)	0.0021		1.55 (0.81-2.96)	0.18	2.23 (1.23-4.05)	0.0082
182	N	0.489	0.507	0.93 (0.60-1.44)	0.74		0.71 (0.45-1.13)	0.15	1.19 (0.75-1.89)	0.46
182	S	0.511	0.487	1.10 (0.71-1.70)	0.67		1.42 (0.90-2.25)	0.13	0.86 (0.54-1.37)	0.53
185	I	0.456	0.395	1.28 (0.83-1.98)	0.27		0.84 (0.52-1.36)	0.48	1.60 (1.01-2.53)	0.047
185	T	0.544	0.599	0.80 (0.52-1.24)	0.32		1.21 (0.75-1.94)	0.44	0.64 (0.40-1.02)	0.060
197	N	0.122	0.199	0.56 (0.29-1.07)	0.081		0.92 (0.46-1.86)	0.82	0.62 (0.32-1.19)	0.15
197	S	0.878	0.795	1.85 (0.97-3.56)	0.064		1.13 (0.56-2.27)	0.74	1.68 (0.87-3.24)	0.12
203	I	0.611	0.701	0.67 (0.43-1.05)	0.082		0.69 (0.44-1.09)	0.11	0.91 (0.55-1.49)	0.71
203	V	0.389	0.286	1.59 (1.02-2.49)	0.042		1.52 (0.96-2.40)	0.073	1.18 (0.71-1.94)	0.52
220	H	0.489	0.501	0.95 (0.62-1.47)	0.82		0.73 (0.46-1.15)	0.17	1.22 (0.76-1.94)	0.41
220	R	0.511	0.485	1.11 (0.72-1.72)	0.64		1.43 (0.91-2.26)	0.12	0.87 (0.54-1.38)	0.55
221	H	0.489	0.501	0.95 (0.62-1.47)	0.82		0.73 (0.46-1.15)	0.17	1.22 (0.76-1.94)	0.41
221	Q	0.511	0.485	1.11 (0.72-1.72)	0.64		1.43 (0.91-2.26)	0.12	0.87 (0.54-1.38)	0.55
224	Q	0.867	0.851	1.14 (0.60-2.15)	0.69		0.67 (0.34-1.32)	0.24	1.02 (0.54-1.95)	0.94
224	R	0.133	0.135	0.98 (0.52-1.86)	0.96		1.69 (0.85-3.37)	0.13	1.09 (0.57-2.07)	0.80
230	A	0.822	0.751	1.54 (0.88-2.70)	0.13		0.87 (0.47-1.64)	0.67	1.37 (0.78-2.43)	0.28
230	P	0.178	0.244	0.67 (0.38-1.18)	0.17		1.18 (0.63-2.22)	0.60	0.75 (0.42-1.33)	0.32

* Fisher's exact test was used for association analysis, otherwise logistic regression was used.

Table S4. Results of the HLA amino acid association analysis (neuromyelitis optica spectrum disorder)

Variant			Frequency				
	Position	amino acid	NMO (n=31)	Control (n=429)	OR(95%CI)	*P*	Fisher
**HLA-DQα1**	-16	L	0.516	0.540	0.91 (0.54-1.52)	0.72	
**(90 amino acid)**	-16	M	0.484	0.460	1.10 (0.66-1.84)	0.72	
	-13	A	0.903	0.948	0.52 (0.21-1.26)	0.15	
	-13	T	0.097	0.052	1.94 (0.79-4.73)	0.15	
	-7	M	0.032	0.066	0.47 (0.05-1.85)	0.42	*
	-7	V	0.968	0.934	2.13 (0.51-8.96)	0.30	
	-6	M	0.952	0.853	3.39 (1.05-11.0)	0.042	
	-6	T	0.048	0.147	0.30 (0.06-0.93)	0.035	*
	2	D	0.968	0.934	2.13 (0.51-8.96)	0.30	
	2	G	0.032	0.066	0.47 (0.05-1.85)	0.42	*
	11	C	0.484	0.485	1.00 (0.59-1.67)	0.99	
	11	Y	0.516	0.515	1.00 (0.60-1.68)	0.99	
	18	F	0.484	0.485	1.00 (0.59-1.67)	0.99	
	18	S	0.516	0.515	1.00 (0.60-1.68)	0.99	
	25	F	0.177	0.226	0.74 (0.38-1.44)	0.38	
	25	Y	0.823	0.774	1.35 (0.69-2.65)	0.38	
	26	S	0.290	0.377	0.68 (0.38-1.19)	0.18	
	26	T	0.710	0.624	1.48 (0.84-2.60)	0.18	
	34	E	0.452	0.502	0.82 (0.49-1.37)	0.44	
	34	Q	0.548	0.498	1.23 (0.73-2.06)	0.44	
	40	E	0.774	0.863	0.55 (0.29-1.02)	0.059	
	40	G	0.226	0.138	1.83 (0.98-3.42)	0.059	
	41	K	0.177	0.204	0.84 (0.43-1.65)	0.62	
	41	R	0.823	0.796	1.19 (0.61-2.33)	0.62	
	45	A	0.484	0.485	1.00 (0.59-1.67)	0.99	
	45	V	0.516	0.515	1.00 (0.60-1.68)	0.99	
	47	C	0.226	0.138	1.83 (0.98-3.42)	0.059	
	47	Q	0.290	0.377	0.68 (0.38-1.19)	0.18	
	47	R	0.484	0.485	1.00 (0.59-1.67)	0.99	
	48	L	0.516	0.515	1.00 (0.60-1.68)	0.99	
	48	W	0.484	0.485	1.00 (0.59-1.67)	0.99	
	50	E	0.484	0.485	1.00 (0.59-1.67)	0.99	
	50	L	0.290	0.378	0.67 (0.38-1.19)	0.17	
	50	V	0.226	0.138	1.83 (0.98-3.42)	0.059	
	51	F	0.774	0.863	0.55 (0.29-1.02)	0.059	
	51	L	0.226	0.138	1.83 (0.98-3.42)	0.059	
	52	R	0.516	0.514	1.01 (0.60-1.69)	0.97	
	52	S	0.484	0.485	1.00 (0.59-1.67)	0.99	
	53	K	0.484	0.485	1.00 (0.59-1.67)	0.99	
	53	Q	0.226	0.138	1.83 (0.98-3.42)	0.059	
	53	R	0.290	0.378	0.67 (0.38-1.19)	0.17	
	55	G	0.484	0.485	1.00 (0.59-1.67)	0.99	
	55	R	0.516	0.515	1.00 (0.60-1.68)	0.99	
	56	G	0.484	0.485	1.81 (0.97-3.39)	0.063	
	56	O	0.226	0.139	1.00 (0.59-1.67)	0.99	
	56	R	0.290	0.377	0.68 (0.38-1.19)	0.18	
	61	F	0.516	0.515	1.00 (0.60-1.68)	0.99	
	61	G	0.484	0.485	1.00 (0.59-1.67)	0.99	
	64	R	0.484	0.485	1.00 (0.59-1.67)	0.99	
	64	T	0.516	0.515	1.00 (0.60-1.68)	0.99	
	66	I	0.516	0.515	1.00 (0.60-1.68)	0.99	
	66	M	0.484	0.485	1.00 (0.59-1.67)	0.99	
	69	A	0.484	0.485	1.00 (0.59-1.67)	0.99	
	69	L	0.484	0.460	1.10 (0.66-1.84)	0.72	
	69	T	0.032	0.055	0.58 (0.07-2.29)	0.77	*
	75	I	0.807	0.917	0.38 (0.19-0.74)	0.0045	
	75	S	0.194	0.083	2.66 (1.35-5.23)	0.0045	
	76	L	0.226	0.139	1.81 (0.97-3.39)	0.063	
	76	M	0.484	0.485	1.00 (0.59-1.67)	0.99	
	76	V	0.290	0.377	0.68 (0.38-1.19)	0.18	
	80	S	0.516	0.515	1.00 (0.60-1.68)	0.99	
	80	Y	0.484	0.485	1.00 (0.59-1.67)	0.99	
	107	I	0.194	0.083	2.66 (1.35-5.23)	0.0045	
	107	T	0.807	0.917	0.38 (0.19-0.74)	0.0045	
	129	H	0.694	0.719	0.88 (0.50-1.55)	0.67	
	129	Q	0.307	0.281	1.13 (0.65-1.98)	0.67	
	130	A	0.177	0.204	0.84 (0.43-1.65)	0.62	
	130	S	0.823	0.796	1.19 (0.61-2.33)	0.62	
	156	F	0.807	0.917	0.38 (0.19-0.74)	0.0045	
	156	L	0.194	0.083	2.66 (1.35-5.23)	0.0045	
	160	A	0.677	0.683	0.97 (0.56-1.69)	0.93	
	160	D	0.226	0.288	0.72 (0.39-1.33)	0.30	
	160	S	0.097	0.029	3.57 (1.41-9.06)	0.0074	
	161	D	0.807	0.917	0.38 (0.19-0.74)	0.0045	
	161	E	0.194	0.083	2.66 (1.35-5.23)	0.0045	
	163	I	0.807	0.917	0.38 (0.19-0.74)	0.0045	
	163	S	0.194	0.083	2.66 (1.35-5.23)	0.0045	
	175	E	0.323	0.432	0.63 (0.36-1.08)	0.094	
	175	K	0.194	0.083	2.66 (1.35-5.23)	0.0045	
	175	Q	0.484	0.485	1.00 (0.59-1.67)	0.99	
	187	A	0.710	0.624	1.48 (0.84-2.60)	0.18	
	187	T	0.290	0.377	0.68 (0.38-1.19)	0.18	
	199	A	0.968	0.937	2.01 (0.48-8.46)	0.34	
	199	T	0.032	0.063	0.50 (0.06-1.97)	0.58	*
	207	M	0.145	0.156	0.92 (0.44-1.90)	0.82	
	207	V	0.855	0.844	1.09 (0.53-2.26)	0.82	
	215	F	0.710	0.622	1.48 (0.84-2.61)	0.17	
	215	L	0.290	0.378	0.67 (0.38-1.19)	0.17	
	218	Q	0.484	0.485	1.00 (0.59-1.67)	0.99	
	218	R	0.516	0.515	1.00 (0.60-1.68)	0.99	

* Fisher's exact test was used for association analysis, otherwise logistic regression was used.
